# Association between the use of β-adrenergic receptor blockers and all-cause mortality in sepsis-associated rhabdomyolysis syndrome: a cohort study

**DOI:** 10.3389/fmed.2026.1743813

**Published:** 2026-02-13

**Authors:** Xiaona Yi, Shanshan Huang, Meixia Zheng, Xingkai Shen, Shaofeng Jin, Zengmin Dai, Yuhong Jin

**Affiliations:** Department of Critical Care Medicine, Ningbo Medical Center LiHuiLi Hospital (The Affiliated LiHuiLi Hospital of Ningbo University), Ningbo, Zhejiang, China

**Keywords:** mimic, mortality, propensity score matching, sepsis-associated rhabdomyolysis, β-blockers

## Abstract

**Background:**

To assess the association between the use of β-blockers and all-cause mortality in Sepsis-associated Rhabdomyolysis (SAR).

**Methods:**

This retrospective cohort study involves adults with SAR. Study variables were extracted from the Medical Information Mart for Intensive Care-IV (MIMIC-IV) database. Propensity score matching (PSM) was conducted at a 1:1 ratio to analyze the association between the use of β-blockers and in-hospital mortality in SAR. Multivariable analysis was employed to adjust for confounding factors, while sensitivity analysis and subgroup analysis were conducted to demonstrate the robustness of the results.

**Results:**

This study involved pre-matched and propensity score-matched cohorts comprising 1,194 and 584 patients, respectively. Through propensity score matching (PSM) analysis, this study observed a notable difference in in-hospital mortality rates. Importantly, the utilization of β-blockers was found to be significantly associated with lower in-hospital all-cause mortality. Furthermore, sensitivity analyses conducted on the entire cohort, as well as cohorts excluding patients with specific comorbidities, consistently demonstrated a significant association between β-blocker usage and lower in-hospital mortality. Subgroup analyses further underscored the robustness of the findings.

**Conclusions:**

The use of β-blockers was associated with lower mortality in patients with SAR. However, prospective studies are needed to validate this finding.

## 1 Introduction

Sepsis is a life-threatening organ dysfunction resulting from a dysregulated host inflammatory response and represents a major global public health burden due to its high mortality, disability, and healthcare costs (1–4). Among its complications, rhabdomyolysis has gained increasing attention, particularly in patients with severe infections. This condition is characterized by the breakdown of skeletal muscle and the subsequent release of myoglobin and other intracellular components into the circulation (5), with reported mortality rates ranging from 1.7% to 46% (6). The interaction between sepsis and rhabdomyolysis is bidirectional, as each condition can exacerbate the other, and patients with sepsis-associated rhabdomyolysis (SAR) are at 39.3% risk for adverse outcomes, including an in-hospital mortality (7). Notably, up to 50% of septic patients with rhabdomyolysis may develop acute kidney injury (AKI) (8), underscoring the importance of early recognition and timely management. Together, these findings highlight the substantial clinical burden of SAR and the need for improved understanding of its prognostic implications.

SAR is increasingly recognized in critically ill patients and is associated with adverse clinical outcomes. Epidemiological data suggest that infectious diseases, including sepsis, account for approximately 19.8% of severe rhabdomyolysis cases (9). Infection-induced metabolic disturbances and inflammatory responses contribute to muscle injury, culminating in myocyte necrosis (10–13). Both viral pathogens and bacterial infections can induce rhabdomyolysis through mechanisms including direct myocyte injury, tissue hypoxia, impaired energy metabolism, endotoxin-mediated metabolic myopathy, and calcium overload (14–16). These processes substantially increase the risk of AKI, poor prognosis, and mortality (17).

β-Adrenergic blockers have attracted growing interest in sepsis management due to their ability to modulate excessive sympathetic activation. By attenuating catecholamine surges, β-blockers may help optimize myocardial oxygen balance and exert beneficial effects on the inflammatory response (18). The impact of β-blockers on mortality in sepsis remains debated. A meta-analysis suggested that β-blockers in septic patients may improve outcomes, including lower mortality (7). However, another study cautions that β-blockers could worsen hemodynamic instability, particularly in severe sepsis or septic shock (19). The pathophysiological of SAR may be driven by factors such as tissue hypoxia, metabolic imbalance, inflammatory cytokines, and direct myocyte injury (20). β-blockers may be associated with a lower adverse prognosis in SAR by reducing inflammation and improving tissue oxygenation (21). However, studies on the association between β-blocker use and mortality in SAR remain limited and inconclusive.

Therefore, this study aims to evaluate the association between β-blocker use and all-cause mortality in patients with SAR. The study employed propensity score matching (PSM) to balance baseline characteristics and minimize confounding factors, thereby ensuring robust and reliable comparisons between groups.

## 2 Methods

This retrospective cohort study utilized data from the MIMIC-IV (v2.2) database, managed by the Massachusetts Institute of Technology (MIT). The dataset includes adult ICU patients with SAR admitted to Beth Israel Deaconess Medical Center between 2008 and 2019. The study adhered to STROBE guidelines (22). As the dataset consists of de-identified public data, the Institutional Review Boards of MIT and Beth Israel Deaconess Medical Center waived the requirement for informed consent. The first author, having completed the ‘data or specimen-only research' training (ID: 52390976), is authorized to access this dataset. The study complies with the Declaration of Helsinki (2013 revision), and ethical review was waived by the Institutional Review Board (ID: KY2023ML079) due to the use of public data.

### 2.1 Study design and population

**Figure 1:**
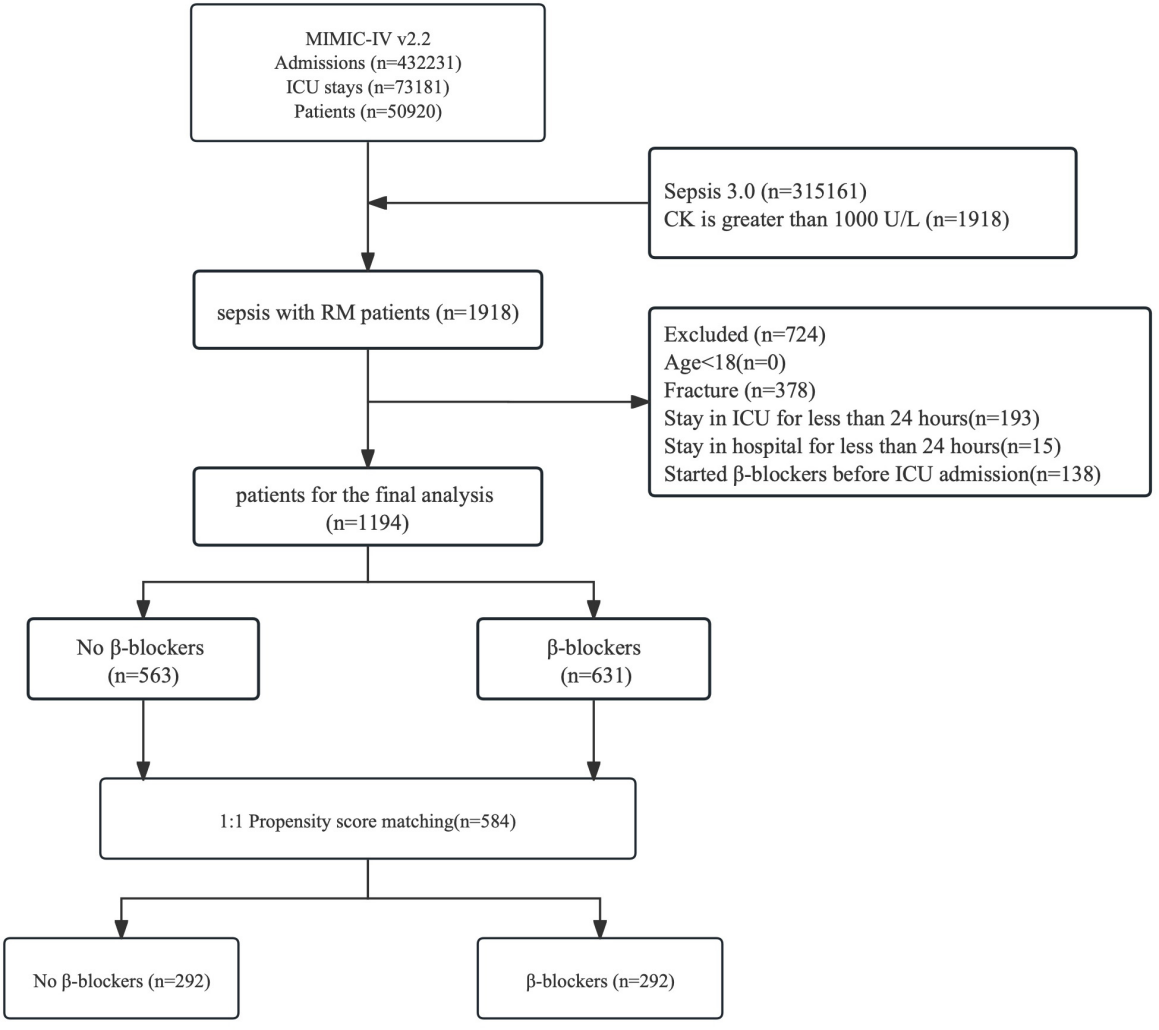
Study flowchart. MIMIC, medical information mart for intensive care; RM, rhabdomyolysis; CK, creatine kinase; SAR, sepsis-associated rhabdomyolysis.

Among the 432,231 admissions recorded in the MIMIC-IV database, 73,181 involved ICU admissions, with 50,920 being first-time ICU admissions. This study focused on patients aged 18 and older diagnosed with SAR based on the MIMIC-IV (v2.2) database. According to the “Rhabdomyolysis: An American Association for the Surgery of Trauma Critical Care Committee Clinical Consensus Document” (23), rhabdomyolysis is defined as creatine kinase (CK) levels exceeding five times the upper limit of normal or over 1,000 U/L. Due to CK typically reaches its peak 24–72 h after muscle injury (24), this study enrolled patients diagnosed with sepsis who developed rhabdomyolysis within 3 days of diagnosis. Sepsis is defined according to the Sepsis-3 criteria or an increased Sequential Organ Failure Assessment (SOFA) score of ≥2. The detailed exclusion criteria are outlined in Figure 1.

Exposure to β-blockers was defined as a prescription containing a β-blocker during the ICU stay. The β-blockers included “Acebutolol,” “Atenolol,” “Esmolol,” “Betaxolol,” “Bisoprolol,” “Labetalol,” “Metoprolol,” “Nadolol,” and “Propranolol.” The routes of administration of β-blocker include intravenous push and oral administration.

Eligible patients were classified into two groups according to β-blocker exposure: those who received β-blocker use after ICU admission (β-blocker group) and those who did not receive β-blockers during their ICU stay (non-β-blocker group).

### 2.2 Covariates and outcomes

The covariables were selected based on clinical *a priori* knowledge as well as their statistical relevance. Covariates with initial regression coefficient changes exceeding 10% were selected by stepwise adjustments in the basic and full models. Collinearity was assessed using the variance inflation factor (VIF), with $\sqrt{VIF}$ > 2 indicating collinearity (25, 26). Details on covariates and missing data rates are provided in Supplementary Table 1 and Supplementary Figure 1. Missing data were addressed using multiple imputations via chained equations prior to propensity score matching. All variables presented in Supplementary Table 2 were incorporated into the PSM procedure.

The primary outcome assessed was all-cause in-hospital mortality, while secondary outcomes included 28-day mortality, 90-day mortality, and ICU mortality.

### 2.3 Statistical analysis

Baseline characteristics are presented as mean ± standard deviation (SD) or median and interquartile range (IQR) for continuous variables, and as number (percentage) for categorical variables. The normality of continuous variables was assessed using Student's *t*-test or Wilcoxon rank-sum test. Between-group differences in categorical variables were evaluated using Pearson's chi-square test or Fisher's exact test.

To explore the association between β-blocker use and in-hospital mortality, we employed PSM to adjust for potential confounders (Supplementary Figures 2, 3). Variables included in the PSM were selected based on a combination of clinical *a priori* knowledge and their statistical relevance to both exposure and outcome. To ensure comprehensive adjustment for baseline differences, all baseline covariates presented in Supplementary Table 2 were incorporated into the propensity score estimation. This approach allowed us to account for clinically meaningful confounders while minimizing residual confounding.

For the PSM analysis, propensity scores (PS) were estimated using a logistic regression model that incorporated all baseline variables listed in Supplementary Table 2. These variables were selected based on clinical *a priori* relevance and their potential statistical association with both treatment assignment and outcomes. Patients in the β-blocker and non-β-blocker groups were matched 1:1 using nearest neighbor matching with a caliper width of 0.1 SD or less. Covariate balance before and after matching was assessed using the absolute standardized mean difference (SMD), with an SMD > 0.1 indicating residual imbalance.

To rigorously estimate the association between β-blocker use and clinical outcomes while minimizing confounding bias, we employed a series of analytical models based on propensity scores (PS). Full Cohort Analyses: Using the entire sample, we performed (a) a PS-adjusted model (including PS as a covariate), (b) Logistic regression in the 1:1 PS-matched cohort, (c) an inverse probability of treatment weighting (IPTW) analysis to balance baseline covariates. The PS was estimated using a multivariable logistic regression model. Matched cohort: Within matched cohort, we performed, (d) a crude logistic regression, and (e) a multivariable-adjusted model for residual confounding. All results are reported as odds ratios (ORs) with 95% confidence intervals (CIs) and are presented together in Table 1.

**Table 1:** Association between β-blocker use and mortality in SAR.

**Models**	**OR (95% CI)**	***p* value**
Adjust for propensity score^a^	0.49 (0.35–0.69)	<0.001
Matched for propensity score^b^	0.56 (0.38–0.84)	0.005
Weighted. IPTW^c^	0.56 (0.43–0.72)	<0.001
In-hospital mortality		
Crude analysis^d^	0.41 (0.31–0.55)	<0.001
Multivariable analysis^e^	0.32 (0.18–0.56)	<0.001
ICU mortality		
Crude analysis	0.52 (0.34–0.8)	0.003
Multivariable analysis	0.43 (0.26–0.74)	0.002
28-day mortality		
Crude analysis	0.59 (0.4–0.88)	0.01
MMultivariable analysis	0.53 (0.33–0.86)	0.01
90-day mortality		
Crude analysis	0.52 (0.36–0.77)	0.001
Multivariable analysis	0.42 (0.27–0.67)	<0.001

OR, odds ratio; CI, confidence interval; PS, propensity score; PSM, propensity score matching; IPTW, inverse probability of treatment weighting.

^a^Logistic regression in the entire (unmatched) cohort with the propensity score included as a covariate.

^b^Logistic regression in the 1:1 PS-matched cohort.

^c^Weighted logistic regression in the entire cohort using inverse probability of treatment weights (IPTW).

^d^Crude logistic regression in matched cohort without further adjustment.

^e^Multivariable logistic regression in matched cohort with further adjustment.

We conducted subgroup analyses to assess the association between β-blocker use and all-cause mortality within subgroups defined by gender, race, age (<65 years or ≥65 years), Sofa, age and sofa, continuous renal replacement therapy (CRRT), mechanical ventilation support, Diabetic, Renal disease, and Chronic pulmonary disease.

To further validate the association between the use of β-blocker and all-cause mortality, we conducted a sensitivity analysis after excluding patients with myocardial infarction, heart failure, cerebrovascular disease, and peripheral vascular disease. Regarding other potential etiologies of rhabdomyolysis, we additionally performed a sensitivity analysis excluding patients with trauma, intoxication, or seizures, which are common non-infectious causes of rhabdomyolysis. To mitigate potential immortal-time bias, we conducted a series of analyses in the full cohort. Landmark analyses were conducted at 24 and 48 h following ICU admission. Patients were stratified according to whether β-blocker use was initiated before or after each predefined landmark. In addition, a time-dependent stratified Cox proportional hazards model was employed to examine the association between β-blocker use and in-hospital mortality. Patients were categorized into three temporal strata based on the timing of β-blocker initiation: within 24 h, between 24–48 h, and beyond 48 h. β-blocker exposure was modeled as a time-dependent covariate, with separate coefficients estimated for each stratum. Furthermore, sensitivity analyses were performed using multivariable Cox regression among patients who received β-blockers within 24 h and those who initiated use within 48 h.

Data analysis was performed using packages R4.1.2 (The R Foundation1) and Free Statistics software version 1.9. Statistical significance was defined as *p* < 0.05.

## 3 Results

### 3.1 Study population and baseline characteristics

Among the 50,920 patients admitted to the ICU for the first time, a total of 1,194 patients were included, with 563 in the non-β-blocker group and 631 in the β-blocker group before matching. After 1:1 propensity score matching, 292 pairs were successfully matched (Supplementary Table 2). Compared to the non-β-blockers group, patients in the β-blockers group were older (64.63 [15.55] years vs. 54.33 [18.19] years), had a higher mean (SD) Charlson comorbidity index score (5.56 [2.62] vs. 3.98 [3.04]), and had longer mechanical ventilation duration (2.16 [2.99] vs. 1.99 [2.93]). However, they had a lower mean sequential organ failure assessment score (6.81 [4.19] vs. 8.2 [4.72]), lower VIS scores (8.04 [23.94] vs. 12.61 [23.83]), lower CRRT) utilization rate (60 [9.5] vs. 83 [14.7]), and lower mechanical ventilation utilization rate (331 [52.5] vs. 359 [63.8]). After 1:1 propensity score matching, the matched cohort included 584 patients, with comparability between the two groups on all specific variables where SMD was less than 0.1 (e.g., mean [SD] age, 59.68 [16.13] years vs. 59.58 [17.39] years; SMD = 0.006). In the overall matched cohort, MICU patients accounted for the largest proportion of ICU admissions (215 [36.8%]); 352 patients (60.27%) required mechanical ventilation support upon ICU admission. Before PSM, there were notable differences in the distribution of infection sites between the β-blocker group and the non-β-blocker group. The standardized mean differences (SMDs) ranged from 0.009 to 0.1, with bacteremia (2.1% vs. 3.7%; SMD = 0.1), pneumonia (30.1% vs. 27.4%; SMD = 0.061), and urinary tract infection (17.9% vs. 15.5%; SMD = 0.066) showing the largest imbalances. After 1:1 propensity score matching, the distributions of infection sites were well-balanced between the matched cohorts, with all SMDs were below 0.1. Specifically, the SMDs for bacteremia (3.4% vs. 4.1%; SMD = 0.036), pneumonia (32.9% vs. 30.5%; SMD = 0.052), and urinary tract infection (19.5% vs. 18.8%; SMD = 0.017) indicated excellent covariate balance after matching. As shown in Supplementary Table 3, before matching, the β-blocker group showed significantly lower in-hospital mortality compared to the non-β-blocker group (15.4% vs. 30.6%, *p* < 0.001). After matching, the β-blocker group continued to demonstrate significantly lower in-hospital mortality (16.8% vs. 26.4%, *p* = 0.009). Similar reductions were observed for 28-day, 90-day mortality and ICU mortality. These findings suggest that β-blocker use is associated with lower mortality in SAR patients even after balancing baseline variables.

In the entire cohort, metoprolol was used significantly more frequently in survivors than in non-survivors (54.1% vs. 33.5%, *p* < 0.001), while atenolol and betaxolol were used exclusively in survivors. In contrast, esmolol was used more frequently in non-survivors (4.5% vs. 1.7%, *p* = 0.009). These trends persisted in the matched cohort (metoprolol: 48.7% vs. 33.3%, *p* = 0.002; esmolol: 6.3% vs. 1.3%, *p* = 0.004). The time to first β-blocker administration was significantly longer in survivors (median: 4.1 vs. 0 h in the entire cohort; 4.4 vs. 0 h in the matched cohort; both *p* < 0.001), and the total duration of medication was also significantly longer (median: 48.0 vs. 0 h in the entire cohort; 38.0 vs. 0 h in the matched cohort; both *p* < 0.001). Regarding the route of administration, oral/nasogastric administration was more common in survivors (28.9% vs. 5.9% in the entire cohort), while intravenous-only administration was more frequent in non-survivors (19.0% vs. 4.5%, *p* < 0.001). In terms of infection sites, bacteremia was observed only in survivors in the matched cohort (5.0% vs. 0%, *p* = 0.007), and urinary tract infections were also significantly more common in survivors (21.4% vs. 11.1%, *p* = 0.009; Supplementary Table 4).

### 3.2 Association between β-blocker use and mortality

In the propensity score–adjusted model, β-blocker use was associated with a significantly lower odds of mortality (OR = 0.49, 95% CI 0.35–0.69; *p* < 0.001). Similar findings were observed in the propensity score–matched cohort, in which the odds of death remained significantly lower among β-blocker users (OR = 0.56, 95% CI 0.38–0.84; *p* = 0.005). The inverse probability of treatment weighting (IPTW) analysis yielded nearly identical results (OR = 0.56, 95% CI 0.43–0.72; *p* < 0.001). The consistency of effect estimates across the adjusted, matched, and weighted models supports the robustness of the association between β-blocker use and lower mortality.

**Figure 2:**
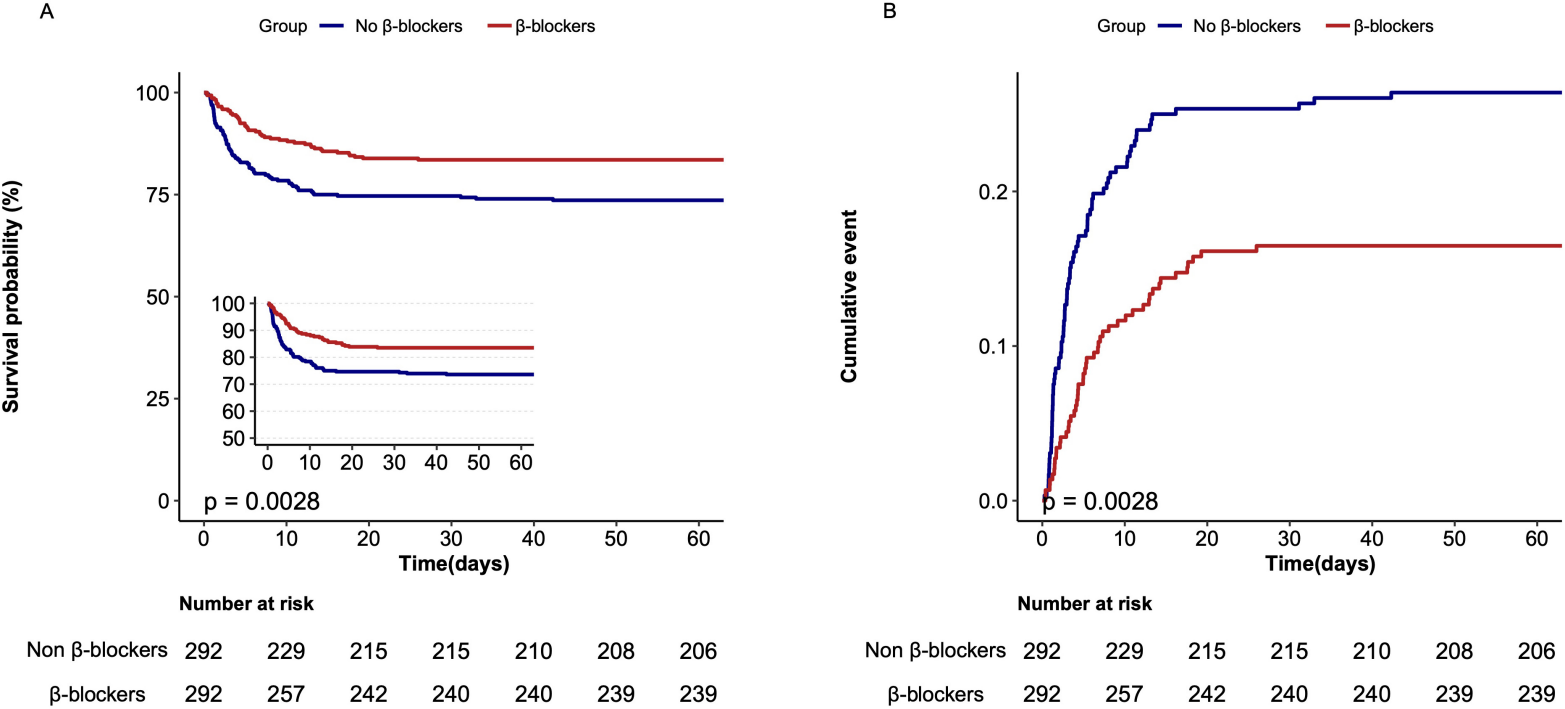
Kaplan–Meier survival analysis curve and cumulative incidence of in-hospital mortality in matched cohort. **(A)** Kaplan–Meier survival analysis curve for β-blockers use. **(B)** Cumulative incidence of in-hospital mortality for β-blockers use.

In the matched cohort, the in-hospital mortality rate was significantly lower in the β-blockers group compared to the non-β-blockers group (49 [16.8%] vs. 77 patients [26.4%]; *p* = 0.005; Table 1, Figure 2). However, patients in the β-blockers group had a longer median (IQR) length of stay (LOS) in the ICU (4.0 [2.2, 7.8] days vs. 3.8 [2.1, 7.1] days; *p* = 0.007) and in the hospital (9.1 [5.7, 15.1] days vs. 7.5 [4.8, 12.7] days; *p* = 0.002; Supplementary Table 1). Univariate logistic regression analysis indicated that the length of ICU stay (LOS ICU) was not significantly associated with mortality (OR, 1.02; 95% CI, 0.99–1.05; *p* = 0.193). In contrast, the length of hospital stay (LOS hospital) was significantly associated with a lower mortality risk (OR, 0.91; 95% CI, 0.88–0.95; *p* < 0.001; Supplementary Table 5). Additionally, ICU mortality, 28-day mortality, and 90-day mortality were all significantly lower in the β-blockers group than in the non-β-blockers group (Table 1, Supplementary Figure 4). In the entire cohort and matched cohort, multivariable Cox regression analysis revealed that patients receiving β-blockers had significantly lower rates of in-hospital mortality, ICU mortality, as well as 28-day and 90-day mortality, compared to those not receiving β-blockers (Table 2, Supplementary Figure 5). Given the potential alternative etiologies of rhabdomyolysis, we conducted a Cox multivariable regression analysis after excluding patients with trauma, intoxication, or seizure to demonstrate the robustness of the findings (Supplementary Table 6). Additionally, a sensitivity analysis, which excluded patients with heart failure and myocardial infarction, confirmed that β-blocker use was associated with lower mortality in patients with SAR (Supplementary Table 7).

**Table 2:** Cox multivariate regression analysis of the association between β-blocker use and mortality.

**Variable**	Entire cohort						Matched cohort					
	*n*. total	*n*. event_%	Unadjusted		Adjusted		*n*. total	*n*. event_%	Unadjusted		Adjusted	
			HR (95% CI)	*p* value	HR (95% CI)	*p* value			HR (95% CI)	*p* value	HR (95% CI)	*p* value
In-hospital mortality												
No β-blockers	563	195 (34.6)	1 (Ref)	<0.001	1 (Ref)	<0.001	292	77 (26.4)	1 (Ref)	0.003	1 (Ref)	<0.001
β-blockers	631	127 (20.1)	0.5 (0.4–0.63)		0.31 (0.24–0.4)		292	49 (16.8)	0.58 (0.41–0.83)		0.51 (0.36–0.75)	
ICU mortality												
No β-blockers	563	195 (34.6)	1 (Ref)	<0.001	1 (Ref)	<0.001	292	68 (23.3)	1 (Ref)	0.002	1 (Ref)	<0.001
β-blockers	631	127 (20.1)	0.5 (0.4–0.63)		0.31 (0.24–0.4)		292	40 (13.7)	0.54 (0.37–0.8)		0.48 (0.32–0.72)	
90-day mortality												
No β-blockers	563	195 (34.6)	1 (Ref)	<0.001	1 (Ref)	<0.001	292	91 (31.2)	1 (Ref)	0.001	1 (Ref)	<0.001
β-blockers	631	127 (20.1)	0.5 (0.4–0.63)		0.34 (0.26–0.44)		292	56 (19.2)	0.56 (0.4–0.78)		0.48 (0.34–0.68)	
28-day mortality												
No β-blockers	563	177 (31.4)	1 (Ref)	<0.001	1 (Ref)	<0.001	292	77 (26.4)	1 (Ref)	0.006	1 (Ref)	0.001
β-blockers	631	97 (15.4)	0.43 (0.33–0.55)		0.25 (0.19–0.33)		292	51 (17.5)	0.61 (0.43–0.86)		0.54 (0.38–0.78)	

HR, hazard ratios; CI, confidence interval.

Entire cohort adjusted for sex, age, ICU type, calcium, activated partial thromboplastin time, myocardial infarct, congestive heart failure, vasoactive-inotropic score, mechanical ventilation.

Matched cohort adjusted for sex, age, ICU type, temperature, activated partial thromboplastin time, myocardial infarct, vasoactive-inotropic score, sodium bicarbonate.

### 3.3 Exploratory subgroup analyses

**Figure 3:**
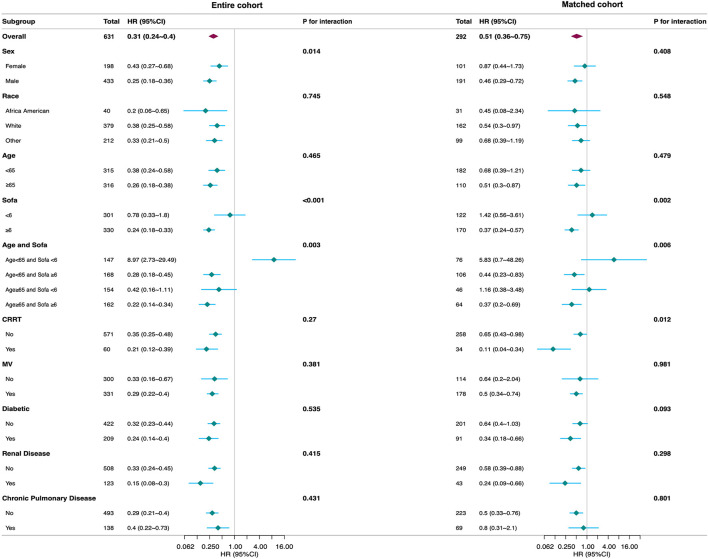
Subgroup analysis of the association between β-blocker use and in-hospital mortality. The stratification factor was not used as an adjustment variable. The small diamonds represent hazard ratios, and the horizontal line represents the 95% confidence interval. The large diamonds represent the overall HRs, whereas the outer points of the diamonds represent a 95% confidence interval. HR, hazard ratio; SOFA, sequential organ failure assessment; CRRT, continuous renal replacement therapy; MV, mechanical ventilation.

In patients with SAR, we performed subgroup analyses to assess the interaction between β-blockers and all-cause mortality, as demonstrated in Figure 3 and Supplementary Table 8. In the entire cohort, the adjusted HR for the primary outcome was 0.31 (95% CI: 0.24–0.40). Significant interactions were observed for SOFA score (*p* < 0.001) and the combined Age–SOFA stratification (*p* = 0.003), but not for age alone (*p* = 0.465). Compared with the subgroup with SOFA <6 (HR: 0.78, 95% CI: 0.33–1.80), the use of β-blockers was associated with lower mortality in the subgroup with SOFA ≥ 6 (HR: 0.24, 95% CI: 0.18–0.33). This pattern was consistent in the combined subgroup, with a significant association observed in patients aged ≥65 years with SOFA scores ≥6 (HR: 0.22, 95% CI: 0.14–0.34). In the matched cohort, the overall effect was attenuated but remained significant (HR: 0.51, 95% CI: 0.36–0.75). Interaction effects for SOFA (*p* = 0.002) and Age–SOFA (*p* = 0.006) persisted, while age alone remained non-significant (*p* = 0.479). In the subgroup with a SOFA score > 6, the use of β-blockers was associated with a lower mortality risk (HR: 0.37, 95% CI: 0.24–0.57). A similar pattern was observed in the combined subgroup analyses, with a significant association identified among patients aged ≥65 years with a SOFA score ≥6 (HR: 0.37, 95% CI: 0.20–0.69). In contrast, no such association was observed in the subgroup with a SOFA score <6 or in the corresponding combined subgroup, and the findings did not reach statistical significance. Given the limited sample size of our cohort, the possibility of bias cannot be excluded. Therefore, larger-scale prospective studies are warranted to validate these observations.

### 3.4 Sensitivity analyses

In landmark analyses at 24 and 48 h, β-blocker use was consistently associated with lower in-hospital mortality (Supplementary Table 9 and Supplementary Figure 6). Before the 24-h landmark, in-hospital mortality rates were 3.5% without β-blocker vs. 1.5% with β-blocker (OR = 0.407, 95% CI 0.188–0.881). At 48 h, the corresponding rates were 12.2% and 3.2% (OR = 0.252, 95% CI 0.155–0.409). Among patients surviving beyond each landmark, β-blocker use remained associated with reduced risk, with ORs of 0.478 (95% CI 0.370–0.617) at 24 h and 0.605 (95% CI 0.453–0.807) at 48 h. In time-dependent stratified Cox models, β-blocker use was evaluated across three temporal strata with adjustment for covariates. β-blocker use conferred significant time-dependent benefit: hazard was reduced by 59% in the early phase (HR, 0.41; *p* = 0.026), by 85% in the intermediate phase (HR, 0.15; *p* < 0.001), and by 61% in the late phase (HR, 0.39; *p* < 0.001; Supplementary Table 10 and Supplementary Figure 7). In the multivariable Cox regression restricted to patients who initiated β-blocker use within 24 h, the adjusted hazard ratios were 0.52 for in-hospital mortality, 0.50 for ICU mortality, 0.49 for 28-day mortality, and 0.52 for 90-day mortality (all *p* < 0.001). In the corresponding analysis limited to β-blocker initiation within 48 h, the association with lower mortality remained consistent, with adjusted hazard ratios ranging from 0.45 to 0.49 across all endpoints (all *p* < 0.001; Supplementary Table 11).

## 4 Discussion

In this study, which included an initial cohort of 1,194 patients and a propensity score-matched cohort of 584 patients, we observed a significant association between β-blocker administration and lower in-hospital mortality among patients with SAR. These findings highlight the potential therapeutic benefits of β-blockers in this high-risk population, warranting further investigation through larger, multicenter trials to confirm these outcomes and explore the underlying mechanisms.

Although our findings indicate a lower mortality associated with β-blocker use in patients with SAR, the underlying biological mechanisms remain incompletely understood. The exact mechanism underlying the association between β-blocker use and lower in-hospital mortality among patients with SAR remains unclear, but several plausible pathways merit consideration. However, it is established that β-blockers can optimize the balance between oxygen supply and demand, which might play a key role in mitigating SAR. The proposed mechanisms for SAR include tissue hypoxia secondary to sepsis, dehydration, toxin release, associated fever, and direct bacterial invasion of muscle (5–8, 27). The use of β-blockers may also be associated with lower mortality through a reduction in excessive exogenous catecholamines (28), although this relationship remains speculative and requires further investigation. Prior studies have suggested that the adjunctive use of selective β1-blockade in septic shock can enhance intrinsic cardiac contractility and vascular responsiveness to catecholamines (29), yet these findings remain associative rather than causal. Taken together, further mechanistic and translational studies are needed to elucidate the pathways underlying the association between β-blocker use and lower mortality.

In our study, consistent with the results of the full cohort analysis, the cohort study using propensity score matching demonstrated that the use of β-blockers is significantly associated with lower in-hospital mortality, ICU mortality, 28-day mortality, and 90-day mortality in patients with SAR. This aligns with Morelli's randomized controlled trial (RCT), which demonstrated that esmolol may be associated with lower mortality by controlling heart rate in patients with septic shock (30). It reported a 28-day mortality of 49.4% in the esmolol group compared with 80.5% in the control group (adjusted hazard ratio, 0.39; 95% CI, 0.26–0.59; *p* < 0.001). Similarly, a multicenter RCT in Japan showed that landiolol effectively lowers heart rate, and reduces new arrhythmias in sepsis-related tachyarrhythmias (31). There were also 12% serious adverse events, including those resulting in death. Coincidentally, a study on sepsis-induced cardiomyopathy (SICM) found that metoprolol can not only improve organ function in sepsis-induced cardiomyopathy (SICM) patients but also associated with lower 28-day mortality (32).

In our cohort, the distribution of specific β-blocker agents differed significantly between survivors and non-survivors, particularly for metoprolol and esmolol, both in the entire cohort and in the matched cohort. Metoprolol was more frequently used among survivors, whereas esmolol showed a higher proportion among non-survivors. These patterns likely reflect underlying clinical decision-making rather than intrinsic differences in drug efficacy, as short-acting agents such as esmolol are often selected for patients with greater hemodynamic instability. This interpretation aligns with prior literature indicating that β-blocker selection in critical illness is strongly influenced by illness severity and the need for rapid titration. Moreover, the low frequency of several β-blocker subtypes (e.g., atenolol, betaxolol, nadolol) limits the ability to draw meaningful comparisons across all agents. Due to the heterogeneous prescribing patterns across β-blocker classes in the ICU setting, our findings underscore that observed differences among subtypes should be interpreted cautiously and are unlikely to represent causal effects.

Contrary to our findings, a UK multicenter RCT involving 40 NHS intensive care units found that landiolol did not improve organ function in septic shock patients with tachycardia treated with norepinephrine for over 24 h (33). In the trial, 28-day mortality was 37.1% in the landiolol group and 25.4% in the standard care group (absolute difference, 11.7% [95% CI, −4.4% to 27.8%]; *p* = 0.16), indicating no statistically significant difference between the two groups. Similarly, 90-day mortality was 43.5% in the landiolol group compared with 28.6% in the standard care group (absolute difference, 15% [95% CI, −1.7% to 31.6%]; *p* = 0.08), again showing no significant mortality benefit associated with landiolol use. This discrepancy may be due to the small sample size (126 cases), potentially leading to biased results. Additionally, as landiolol is a β1-receptor blocker, its limited efficacy in this context should not be generalized to all β-blockers.

Interestingly, there are currently few reports on SAR, though it is well-established that infections (viral, bacterial, etc.) can induce rhabdomyolysis. Since the COVID-19 pandemic, numerous studies have highlighted the link between COVID-19 and rhabdomyolysis (34–37), which is significantly associated with increased ICU admissions and in-hospital mortality (38). COVID-19-related rhabdomyolysis also correlates with higher risks of renal replacement therapy and mortality (39). A 3-year prospective study identified Gram-positive bacteria as the primary pathogens causing rhabdomyolysis in bacterial sepsis, with Gram-negative bacteria being less common (14). Additionally, a retrospective cohort study confirmed that SAR is linked to high mortality (17), with the lungs (40, 41) being the most frequent infection site, followed by the urinary tract, cholecystitis, pancreatitis, and catheter-related infections. However, these studies do not include research on β-blockers in patients with SAR. Our survival analysis showed that SAR patients in the β-blocker group had significantly higher survival rates compared to those not receiving β-blockers.

In our study, plasma calcium ion concentrations were higher in the β-blocker group than in the non-β-blocker group. An observational study has suggested that β-blockers or calcium channel blockers can improve mortality rates in critically ill patients (42). It is known that one of the mechanisms of SAR is the accumulation of intracellular calcium ions, which disrupts cellular homeostasis and ultimately leads to cell death. Our findings indirectly support that in rhabdomyolysis, calcium ions shift intracellularly, with excessive accumulation causing cell lysis and necrosis. However, these conclusions require further validation through large-scale prospective studies.

The observed association between longer hospital or ICU length of stay (LOS) and lower mortality should be interpreted with caution. While patients who survive naturally accumulate longer LOS, those who die early often have shorter stays, introducing potential collider bias and reverse causality. Therefore, LOS may reflect survival duration rather than influence it. Consequently, LOS should be regarded as a descriptive result of disease progression rather than a determinant of prognosis. Thus, larger prospective studies are necessary to validate these results.

Considering the cardioprotective effects of β-blockers in conditions such as heart failure, myocardial infarction, and cardiovascular diseases, we excluded these patient populations and conducted a sensitivity analysis. The results consistently demonstrated that β-blocker use is associated with lower mortality in SAR. Subgroup analyses further supported these findings, except in patients undergoing CRRT, where an interaction was observed. The interaction likely arises from CRRT's ability to effectively remove various molecular sizes, enhancing the management of SAR (43, 44).

In our subgroup analysis, we found that among patients with higher SOFA scores, the use of β-blockers was associated with lower mortality, while age showed no clear correlation. This association appeared even stronger in older patients with high SOFA scores. Because these subgroups were small and had wide confidence intervals (CI), the estimates were unstable. Therefore, the seemingly harmful effect observed in younger patients with lower SOFA scores should be interpreted with caution. Residual confounding or random variation is a more plausible explanation, especially given that the association was not statistically significant after matching. Overall, the relationship between β-blocker use and mortality across age and SOFA score subgroups still require confirmation through large-scale prospective studies.

In this retrospective study, several limitations should be acknowledged. First, selection bias and variability in treatment standards may be inherent to the retrospective study design. To mitigate these biases, we employed propensity score matching and multivariate Cox proportional hazards regression analysis. Second, recognizing the protective role of β-blockers in myocardial infarction and heart failure, we conducted a sensitivity analysis excluding these patients, which confirmed the stability of our findings. Third, the relatively not large sample size and the retrospective nature of the study led to incomplete data, necessitating the exclusion of many samples due to stringent inclusion and exclusion criteria. A further limitation of this study is the lack of consistent documentation regarding the interruption of β-blocker use. Because the retrospective medical records did not reliably capture whether treatment was temporarily withheld or the clinical reasons for discontinuation, we were unable to systematically evaluate the frequency, timing, or causes of therapy interruption. This incomplete information may have constrained our ability to fully interpret the patterns of β-blocker exposure. Moreover, the observed protective association between β-blocker use and clinical outcomes should be interpreted with caution. In clinical practice, β-blockers are often withheld in patients with unstable hemodynamics and continued in those perceived to be at lower risk. This may introduce indication bias, whereby the apparent benefit reflects underlying differences in patient stability rather than a true pharmacologic effect. To address this, we incorporated key hemodynamic variables into our models and performed sensitivity analyses using multiple adjustment strategies. Our initial exposure definition may have introduced immortal time bias; however, the concordant findings from time-dependent and landmark analyses-despite modest shifts in effect estimates-support the robustness of the association after methodological correction. Nonetheless, these observational data cannot establish causality, and residual confounding cannot be excluded. Prospective studies or trial emulation approaches are needed to confirm causality and to determine the optimal timing, dosage, and formulation of β-blocker use in this population.

## 5 Conclusions

The cohort study suggests a potential association between β-blockers use and lower mortality in patients with SAR. However, these findings suggest a potential benefit, but do not establish causality. Further prospective research is needed to confirm these associations and explore the underlying mechanisms.

## Data Availability

The raw data supporting the conclusions of this article will be made available by the authors, without undue reservation.
