# Supplementary material for: Association between the use of β-adrenergic receptor blockers and all-cause mortality in sepsis-associated rhabdomyolysis syndrome: a cohort study
Source: Front Med (Lausanne). 2026 Feb 13;13:1743813. doi: 10.3389/fmed.2026.1743813 (PMC12946102; doi:10.3389/fmed.2026.1743813)
Supplement: Supplementary file 1 [file Table_1.docx]

**Supplementary Table 1. Covariate screening of multicollinearity**

| **Term** | **Entire cohort** | | | | | | | |  | **Matched cohort** | | | | | | | |
| --- | --- | --- | --- | --- | --- | --- | --- | --- | --- | --- | --- | --- | --- | --- | --- | --- | --- |
|  | **Change.% 1** | **Change.% 2** | **VIF** | **DF** | $\sqrt{\text{VIF}}$ | **colinearity** | **select** | **select.VIF** |  | **Change.% 1** | **Change.% 2** | **VIF** | **DF** | $\sqrt{\text{VIF}}$ | **colinearity** | **select** | **select.VIF** |
| Crude | Crude Ref. | FullRef. | 1.58 | 1 | 1.257 | 0 | Ref. | Ref. |  | Crude Ref. | Full Ref. | 1.652 | 1 | 1.285 | 0 | Ref. | Ref. |
| Sex | -0.3 | -1 | 1.681 | 1 | 1.297 | 0 | No | No |  | -0.1 | -3.1 | 2.01 | 1 | 1.418 | 0 | No | No |
| Age | 27.9 | -7.5 | 3.298 | 1 | 1.816 | 0 | Yes | Yes |  | 6 | 2.2 | 3.782 | 1 | 1.945 | 0 | No | No |
| Race | -2.4 | -0.8 | 2.263 | 2 | 1.226 | 0 | No | No |  | -2.4 | -1.9 | 3.584 | 2 | 1.376 | 0 | No | No |
| BMI | 0.2 | -1.1 | 1.505 | 1 | 1.227 | 0 | No | No |  | -2.3 | -4 | 2.018 | 1 | 1.421 | 0 | No | No |
| LosICU | 4 | -8.1 | 10.208 | 1 | 3.195 | 1 | No | Pending |  | 0.3 | 5.3 | 13.006 | 1 | 3.606 | 1 | No | Pending |
| LosHospital | -6.6 | 31.5 | 8.816 | 1 | 2.969 | 1 | Yes | Pending |  | -16.4 | 31.7 | 11.696 | 1 | 3.42 | 1 | Yes | Pending |
| ICUtype | 42.7 | 1.5 | 9.183 | 4 | 1.319 | 0 | Yes | Yes |  | 20.7 | 12 | 23.928 | 4 | 1.487 | 0 | Yes | Yes |
| YearofAdmission | -4.1 | 0.3 | 3.233 | 3 | 1.216 | 0 | No | No |  | 1.7 | -0.2 | 7.432 | 3 | 1.397 | 0 | No | No |
| Heartrate | -0.2 | -2 | 1.986 | 1 | 1.409 | 0 | No | No |  | -1 | -2.6 | 3.162 | 1 | 1.778 | 0 | No | No |
| SBP | 6.5 | -0.4 | 3.336 | 1 | 1.827 | 0 | No | No |  | 6.2 | -4.8 | 3.532 | 1 | 1.879 | 0 | No | No |
| DBP | 3.3 | 0.2 | 3.766 | 1 | 1.941 | 0 | No | No |  | 5.6 | -0.3 | 3.378 | 1 | 1.838 | 0 | No | No |
| MBP | 1.6 | -1.6 | 4.72 | 1 | 2.172 | 1 | No | Pending |  | 5.3 | -0.1 | 4.081 | 1 | 2.02 | 1 | No | Pending |
| Resprate | -4.6 | 0.6 | 1.709 | 1 | 1.307 | 0 | No | No |  | 0.8 | -1.1 | 1.99 | 1 | 1.411 | 0 | No | No |
| Temperature | 8 | 0.8 | 2.063 | 1 | 1.436 | 0 | No | No |  | 12.5 | -0.2 | 3.335 | 1 | 1.826 | 0 | Yes | Yes |
| SpO2 | -3.3 | -2.5 | 2.528 | 1 | 1.59 | 0 | No | No |  | 4.9 | -0.7 | 2.802 | 1 | 1.674 | 0 | No | No |
| Hematocrit | -0.6 | 3.3 | 27.343 | 1 | 5.229 | 1 | No | Pending |  | 0 | -8.6 | 39.177 | 1 | 6.259 | 1 | No | Pending |
| Hemoglobin | -2.5 | 1.8 | 28.648 | 1 | 5.352 | 1 | No | Pending |  | 0.6 | -6 | 40.471 | 1 | 6.362 | 1 | No | Pending |
| Platelets | -1.2 | 0 | 2.423 | 1 | 1.557 | 0 | No | No |  | 1.9 | -3.4 | 3.205 | 1 | 1.79 | 0 | No | No |
| WBC | -2.7 | -0.8 | 1.673 | 1 | 1.293 | 0 | No | No |  | -3.3 | 0.1 | 2.232 | 1 | 1.494 | 0 | No | No |
| Albumin | -1 | -2.9 | 2.735 | 1 | 1.654 | 0 | No | No |  | 7.1 | 0.6 | 3.446 | 1 | 1.856 | 0 | No | No |
| Bicarbonate | -25.9 | 0.8 | 5.165 | 1 | 2.273 | 1 | Yes | Pending |  | 2.6 | 5.9 | 6.846 | 1 | 2.616 | 1 | No | Pending |
| BUN | -8.8 | 2.7 | 5.188 | 1 | 2.278 | 1 | No | Pending |  | 0.5 | 10 | 4.963 | 1 | 2.228 | 1 | Yes | Pending |
| Creatinine | -4.7 | 0 | 3.735 | 1 | 1.933 | 0 | No | No |  | -0.3 | -2.1 | 4.717 | 1 | 2.172 | 1 | No | Pending |
| Calcium | -10.2 | -0.3 | 2.462 | 1 | 1.569 | 0 | Yes | Yes |  | 0 | -0.5 | 3.119 | 1 | 1.766 | 0 | No | No |
| Chloride | -3.3 | -1.2 | 4.081 | 1 | 2.02 | 1 | No | Pending |  | -0.4 | -1 | 4.522 | 1 | 2.127 | 1 | No | Pending |
| INR | 1.1 | 0.7 | 6.648 | 1 | 2.578 | 1 | No | Pending |  | 5.1 | 0.3 | 353.528 | 1 | 18.802 | 1 | No | Pending |
| PT | -0.4 | 1.2 | 7.169 | 1 | 2.678 | 1 | No | Pending |  | 5.8 | 0.6 | 368.805 | 1 | 19.204 | 1 | No | Pending |
| PTT | 35.9 | -0.5 | 1.904 | 1 | 1.38 | 0 | Yes | Yes |  | 22.3 | -6.9 | 2.777 | 1 | 1.666 | 0 | Yes | Yes |
| BilirubinTotal | -2.8 | -3.6 | 3.097 | 1 | 1.76 | 0 | No | No |  | 9.2 | -2 | 6.738 | 1 | 2.596 | 1 | No | Pending |
| CK | 0.4 | -0.1 | 1.996 | 1 | 1.413 | 0 | No | No |  | 1.5 | 0.2 | 1.818 | 1 | 1.348 | 0 | No | No |
| Lactate | -3 | -9.8 | 5.512 | 1 | 2.348 | 1 | No | Pending |  | 37.8 | -1.7 | 10.057 | 1 | 3.171 | 1 | Yes | Pending |
| pH | -16.4 | -8.6 | 8.23 | 1 | 2.869 | 1 | Yes | Pending |  | 7.2 | 4 | 10.7 | 1 | 3.271 | 1 | No | Pending |
| PO2 | -3.3 | 0 | 1.707 | 1 | 1.307 | 0 | No | No |  | 0.6 | 0.1 | 2.048 | 1 | 1.431 | 0 | No | No |
| PCO2 | -2.9 | -1.9 | 5.302 | 1 | 2.303 | 1 | No | Pending |  | 0 | 1.1 | 7.318 | 1 | 2.705 | 1 | No | Pending |
| Sodium | -1.5 | -2 | 3.754 | 1 | 1.938 | 0 | No | No |  | -0.2 | -1.9 | 4.057 | 1 | 2.014 | 1 | No | Pending |
| Potassium | -0.7 | 0.3 | 1.912 | 1 | 1.383 | 0 | No | No |  | -0.1 | -1.2 | 2.327 | 1 | 1.526 | 0 | No | No |
| Phosphate | -13.7 | -4.9 | 4.259 | 1 | 2.064 | 1 | Yes | Pending |  | 1.5 | -0.3 | 4.446 | 1 | 2.108 | 1 | No | Pending |
| Magnesium | -2.7 | 1.8 | 2.146 | 1 | 1.465 | 0 | No | No |  | 3.4 | 2.7 | 2.051 | 1 | 1.432 | 0 | No | No |
| Glucose | 0.3 | -2.5 | 1.912 | 1 | 1.383 | 0 | No | No |  | 1.5 | 0.3 | 2.461 | 1 | 1.569 | 0 | No | No |
| MyocardialInfarct | 26.4 | 0.2 | 2.115 | 1 | 1.454 | 0 | Yes | Yes |  | 10.7 | -2.9 | 3.422 | 1 | 1.85 | 0 | Yes | Yes |
| CongestiveHeartFailure | 15.5 | 1.3 | 1.951 | 1 | 1.397 | 0 | Yes | Yes |  | 8 | 1.8 | 2.607 | 1 | 1.615 | 0 | No | No |
| PeripheralVascularDisease | 9.7 | -2 | 1.703 | 1 | 1.305 | 0 | No | No |  | 1.3 | -0.4 | 3.487 | 1 | 1.867 | 0 | No | No |
| CerebroVascularDisease | 0.2 | -2.4 | 1.842 | 1 | 1.357 | 0 | No | No |  | -1.7 | -1.8 | 2.447 | 1 | 1.564 | 0 | No | No |
| ChronicPulmonaryDisease | 0.6 | -1.2 | 1.76 | 1 | 1.326 | 0 | No | No |  | 0.4 | -0.8 | 2.35 | 1 | 1.533 | 0 | No | No |
| RenalDisease | 2.8 | 0.1 | 2.108 | 1 | 1.452 | 0 | No | No |  | 1.5 | -2.5 | 2.772 | 1 | 1.665 | 0 | No | No |
| Diabetic | 1.7 | 0.8 | 2.015 | 1 | 1.42 | 0 | No | No |  | 1.7 | 0.6 | 2.43 | 1 | 1.559 | 0 | No | No |
| Liverdiseases | -3.3 | -2.8 | 2.273 | 1 | 1.508 | 0 | No | No |  | 0 | -1.7 | 3.117 | 1 | 1.766 | 0 | No | No |
| charlsoncomorbidityindex | 25.5 | -0.2 | 6.331 | 1 | 2.516 | 1 | Yes | Pending |  | 4.6 | -3.3 | 6.443 | 1 | 2.538 | 1 | No | Pending |
| APSIII | -13.8 | -6.8 | 5.508 | 1 | 2.347 | 1 | Yes | Pending |  | 27.7 | -7.1 | 7.053 | 1 | 2.656 | 1 | Yes | Pending |
| SAPSII | 6.6 | 0.5 | 3.72 | 1 | 1.929 | 0 | No | No |  | 23.4 | 0 | 4.621 | 1 | 2.15 | 1 | Yes | Pending |
| SOFAscore | -15.7 | -1 | 7.903 | 1 | 2.811 | 1 | Yes | Pending |  | 22.7 | 0.1 | 11.218 | 1 | 3.349 | 1 | Yes | Pending |
| Bacteremia | 2.2 | -3.9 | 1.263 | 1 | 1.124 | 0 | No | No |  | 2.2 | 1.6 | 1 | 1 | 1 | 0 | No | No |
| Abdoalinfection | 0.2 | -2.5 | 1.643 | 1 | 1.282 | 0 | No | No |  | 1.8 | -0.2 | 3.557 | 1 | 1.886 | 0 | No | No |
| Pneumonia | 0.2 | -0.1 | 1.655 | 1 | 1.286 | 0 | No | No |  | 0.2 | -1.8 | 2.422 | 1 | 1.556 | 0 | No | No |
| SkinandSoftTissueInfection | -0.1 | 0.4 | 1.436 | 1 | 1.198 | 0 | No | No |  | 1.1 | -0.7 | 1 | 1 | 1 | 0 | No | No |
| Urinarytractinfection | -0.4 | 0.2 | 1.306 | 1 | 1.143 | 0 | No | No |  | 0.6 | 0.2 | 1.554 | 1 | 1.247 | 0 | No | No |
| VIP | 13.6 | 1.5 | 2.078 | 1 | 1.441 | 0 | Yes | Yes |  | 21.5 | -8.8 | 3.826 | 1 | 1.956 | 0 | Yes | Yes |
| CRRT | -1.9 | -0.9 | 2.903 | 1 | 1.704 | 0 | No | No |  | 17.1 | 0.2 | 4.781 | 1 | 2.187 | 1 | Yes | Pending |
| MV | -10.8 | 3.4 | 1.756 | 1 | 1.325 | 0 | Yes | Yes |  | 9.6 | 2.6 | 2.28 | 1 | 1.51 | 0 | No | No |
| MVtime | 0.9 | -0.2 | 2.082 | 1 | 1.443 | 0 | No | No |  | 2.8 | 5.5 | 3.4 | 1 | 1.844 | 0 | No | No |
| Mannitol | -1.7 | -1.4 | 1.537 | 1 | 1.24 | 0 | No | No |  | -0.5 | -0.9 | 2.298 | 1 | 1.516 | 0 | No | No |
| Sodiumbicarbonate | 6.2 | 0.4 | 2.241 | 1 | 1.497 | 0 | No | No |  | 16.3 | -9.9 | 3.792 | 1 | 1.947 | 0 | Yes | Yes |
| Statin | 7.2 | -0.9 | 1.603 | 1 | 1.266 | 0 | No | No |  | 3.5 | 1.9 | 2.195 | 1 | 1.482 | 0 | No | No |
| Calciumbuchong | 0.9 | -0.4 | 1.87 | 1 | 1.367 | 0 | No | No |  | 1.4 | 0.4 | 3.222 | 1 | 1.795 | 0 | No | No |
| MagnesiumSulfatesum | 8.7 | -0.7 | 2.48 | 1 | 1.575 | 0 | No | No |  | 5 | 0.5 | 3.537 | 1 | 1.881 | 0 | No | No |
| Potassiumchloride | 0.5 | 1.1 | 1.368 | 1 | 1.169 | 0 | No | No |  | 0 | 0.6 | 2.103 | 1 | 1.45 | 0 | No | No |
| DCCardioversion | 7.5 | 0 | 1.622 | 1 | 1.274 | 0 | No | No |  | -3.7 | -4.3 | 2.715 | 1 | 1.648 | 0 | No | No |

Abbreviations: SD standard deviation; SMD standardized mean difference; SOFA Sequential Organ Failure Assessment; CCI charlson comorbidity index; SpO_2_ Peripheral capillary oxygen saturation; SBP systolic blood pressure; DBP Diastolic blood pressure; MBP mean arterial pressure; APSIII Acute Physiology Score III; SAPS II Simpliﬁed Acute Physiology Score II; SOFA Sequential Organ Failure Assessment; RBC red blood cell counts; WBC white blood cell counts; T-Bil Bilirubin total; BUN blood urine nitrogen; INR International normalized ratio; PT Prothrombin time; PTT Activated partial thromboplastin time; CRRT Continuous renal replacement therapy; MI Myocardial infarct; CHF Congestive heart failure; PVD Peripheral Vascular Disease; CVD Cerebra Vascular Disease; CPD Chronic pulmonary disease; RD renal disease；SSTI Skin and soft tissues infection; UI Urinary infection; MV mechanical ventilation; VIS The vasoactive-inotropic score was calculated as follows: dopamine dose (in micrograms per kilogram per minute) + dobutamine dose (in micrograms per kilogram per minute) + 100 × epinephrine dose (in micrograms per kilogram per minute) + 10 × milrinone dose (in micrograms per kilogram per minute) + 10000 × vasopressin dose (in international units per kilogram per minute) + 100 × norepinephrine dose (in micrograms per kilogram per minute).
