# Supplementary material for: Association between the use of β-adrenergic receptor blockers and all-cause mortality in sepsis-associated rhabdomyolysis syndrome: a cohort study
Source: Front Med (Lausanne). 2026 Feb 13;13:1743813. doi: 10.3389/fmed.2026.1743813 (PMC12946102; doi:10.3389/fmed.2026.1743813)
Supplement: Supplementary file 2 [file Table_2.docx]

**Supplementary Table 2. Baseline characteristics before and after propensity score matching**

| **Characteristic** | **Before propensity score matching**  **Mean (SD)** | | |  | **After propensity score matching**  **Mean (SD)** | | |
| --- | --- | --- | --- | --- | --- | --- | --- |
|  | Non-β blockers (n=563) | β-blockers (n=631) | SMD |  | Non-β blockers (n=292) | β-blockers (n=292) | SMD |
| Age, y | 54.33 (18.19) | 64.63 (15.55) | 0.608 |  | 59.58 (17.39) | 59.68 (16.13) | 0.006 |
| Sex, n (%) |  |  |  |  |  |  |  |
| Male | 365 (64.8) | 433 (68.6) | 0.08 |  | 197 (67.5) | 191 (65.4) | 0.044 |
| Race, n (%) |  |  | 0.202 |  |  |  | 0.05 |
| Africa American | 56 (9.9) | 40 (6.3) |  |  | 30 (10.3) | 31 (10.6) |  |
| White | 286 (50.8) | 379 (60.1) |  |  | 156 (53.4) | 162 (55.5) |  |
| Other | 221 (39.3) | 212 (33.6) |  |  | 106 (36.3) | 99 (33.9) |  |
| Body mass index, kg/m2 | 29.50 (9.04) | 29.13 (6.97) | 0.045 |  | 29.54 (10.03) | 29.51 (7.53) | 0.003 |
| Length of ICU stay, d | 4.87 (5.44) | 6.42 (7.23) | 0.242 |  | 5.50 (6.58) | 6.08 (5.54) | 0.095 |
| Length of hospital stay, d | 9.83 (13.52) | 11.88 (11.43) | 0.164 |  | 11.22 (17.23) | 12.44 (11.95) | 0.082 |
| **ICU type, n (%)** |  |  | 0.728 |  |  |  | 0.08 |
| CCU | 71 (12.6) | 201 (31.9) |  |  | 52 (17.8) | 52 (17.8) |  |
| CVICU | 21 (3.7) | 86 (13.6) |  |  | 18 (6.2) | 21 (7.2) |  |
| MICU | 283 (50.3) | 152 (24.1) |  |  | 111 (38.0) | 104 (35.6) |  |
| SICU | 62 (11.0) | 70 (11.1) |  |  | 38 (13.0) | 44 (15.1) |  |
| Other | 126 (22.4) | 122 (19.3) |  |  | 73 (25.0) | 71 (24.3) |  |
| **Year of Admission, n (%)** |  |  | 0.368 |  |  |  | 0.055 |
| 2008-2010 | 123 (21.8) | 211 (33.4) |  |  | 83 (28.4) | 76 (26.0) |  |
| 2011-2013 | 120 (21.3) | 170 (26.9) |  |  | 73 (25.0) | 76 (26.0) |  |
| 2014-2016 | 157 (27.9) | 139 (22.0) |  |  | 66 (22.6) | 69 (23.6) |  |
| 2017-2019 | 163 (29.0) | 111 (17.6) |  |  | 70 (24.0) | 71 (24.3) |  |
| **Initial vital signs at ICU admission** | | | | | | | |
| Heart rate, bpm | 108.52 (20.01) | 107.81 (21.04) | 0.034 |  | 109.48 (19.89) | 108.38 (20.50) | 0.055 |
| SBP, mm Hg | 88.87 (19.04) | 88.85 (16.41) | 0.001 |  | 90.52 (18.22) | 90.65 (16.88) | 0.007 |
| DBP, mm Hg | 47.39 (13.18) | 47.09 (11.30) | 0.025 |  | 48.75 (12.41) | 48.18 (11.56) | 0.048 |
| MBP, mm Hg | 58.50 (15.05) | 59.13 (13.33) | 0.045 |  | 60.62 (13.40) | 60.52 (13.59) | 0.007 |
| Respiratory rate, bpm | 29.46 (6.71) | 28.11 (5.90) | 0.214 |  | 28.65 (6.53) | 28.68 (6.07) | 0.005 |
| Temperature, °C | 37.57 (1.18) | 37.53 (1.03) | 0.039 |  | 37.65 (1.18) | 37.59 (1.05) | 0.054 |
| SpO2, % | 90.05 (10.06) | 91.29 (6.78) | 0.144 |  | 91.15 (7.57) | 91.23 (7.77) | 0.011 |
| **Laboratory data on day of ICU admission** | | | | | | | |
| Hematocrit, % | 32.61 (6.34) | 33.21 (6.76) | 0.091 |  | 32.76 (6.16) | 32.87 (7.07) | 0.017 |
| Hemoglobin, g/dL | 10.84 (2.15) | 11.23 (2.35) | 0.172 |  | 10.96 (2.09) | 10.99 (2.42) | 0.011 |
| Platelets, ×109/L | 167.36 (78.67) | 176.51 (78.62) | 0.116 |  | 172.60 (80.51) | 171.53 (79.71) | 0.013 |
| WBC, ×109/L | 16.51 (8.29) | 15.74 (7.22) | 0.098 |  | 16.34 (8.85) | 16.12 (7.08) | 0.028 |
| Albumin, g/dL | 3.25 (0.71) | 3.33 (0.68) | 0.111 |  | 3.29 (0.66) | 3.27 (0.68) | 0.028 |
| Bicarbonate, m Eq/L | 18.12 (5.64) | 19.88 (4.49) | 0.344 |  | 18.99 (5.35) | 19.02 (4.79) | 0.007 |
| BUN, mg/dL | 36.38 (32.69) | 29.98 (20.75) | 0.234 |  | 32.74 (25.43) | 32.29 (24.69) | 0.018 |
| Creatinine, mg/dL | 2.38 (2.27) | 1.87 (1.72) | 0.253 |  | 2.21 (2.36) | 2.12 (2.17) | 0.04 |
| Calcium, mg/dL | 7.50 (0.90) | 7.85 (0.92) | 0.383 |  | 7.65 (0.86) | 7.64 (0.92) | 0.007 |
| Chloride, m Eq/L | 107.65 (7.32) | 106.57 (6.03) | 0.162 |  | 106.88 (6.62) | 107.10 (6.86) | 0.033 |
| INR | 1.64 (1.38) | 1.53 (1.14) | 0.09 |  | 1.52 (0.75) | 1.52 (1.04) | 0.008 |
| PT, s | 17.82 (13.75) | 16.76 (11.80) | 0.083 |  | 16.57 (7.76) | 16.56 (10.16) | 0.002 |
| PTT, s | 48.04 (35.59) | 61.94 (43.89) | 0.348 |  | 51.33 (38.53) | 52.73 (39.74) | 0.036 |
| T-Bil, u mol/L | 1.35 (2.64) | 1.13 (1.70) | 0.101 |  | 1.27 (2.52) | 1.27 (2.17) | 0.003 |
| Creatine kinase, mg/dL | 10836.54 (29907.51) | 7513.77 (23926.70) | 0.123 |  | 9832.37 (29367.36) | 9288.84 (27943.42) | 0.019 |
| Lactate, mmol/L | 4.09 (4.25) | 3.33 (2.85) | 0.211 |  | 3.27 (3.14) | 3.41 (3.08) | 0.044 |
| pH | 7.26 (0.14) | 7.30 (0.12) | 0.322 |  | 7.29 (0.12) | 7.29 (0.13) | 0.004 |
| PO2, mm Hg | 88.69 (49.89) | 102.20 (65.31) | 0.233 |  | 98.77 (58.47) | 96.94 (61.86) | 0.03 |
| PCO2, mm Hg | 48.49 (15.39) | 46.65 (13.60) | 0.127 |  | 46.88 (14.96) | 46.83 (12.29) | 0.004 |
| Sodium, m Eq/L | 139.19 (6.44) | 138.11 (5.30) | 0.183 |  | 138.23 (5.74) | 138.59 (6.31) | 0.059 |
| Potassium, m Eq/L | 4.32 (1.00) | 4.27 (0.84) | 0.053 |  | 4.28 (0.94) | 4.27 (0.92) | 0.01 |
| Phosphate, m Eq/L | 4.39 (2.39) | 3.88 (1.61) | 0.249 |  | 4.05 (1.87) | 4.04 (1.89) | 0.008 |
| Magnesium, mg/dL | 2.05 (0.53) | 1.99 (0.45) | 0.118 |  | 1.98 (0.44) | 2.01 (0.46) | 0.058 |
| Glucose, mg/dL | 167.03 (114.78) | 168.77 (85.68) | 0.017 |  | 168.65 (113.24) | 168.12 (93.44) | 0.005 |
| Comorbidities |  |  |  |  |  |  |  |
| MI, n (%) | 120 (21.3) | 314 (49.8) | 0.622 |  | 88 (30.1) | 90 (30.8) | 0.015 |
| CHF, n (%) | 90 (16.0) | 241 (38.2) | 0.516 |  | 73 (25.0) | 80 (27.4) | 0.055 |
| PVD, n (%) | 30 (5.3) | 98 (15.5) | 0.339 |  | 20 (6.8) | 20 (6.8) | <0.001 |
| CVD, n (%) | 82 (14.6) | 99 (15.7) | 0.031 |  | 57 (19.5) | 59 (20.2) | 0.017 |
| CPD, n (%) | 138 (24.5) | 138 (21.9) | 0.063 |  | 69 (23.6) | 69 (23.6) | <0.001 |
| RD, n (%) | 58 (10.3) | 123 (19.5) | 0.26 |  | 43 (14.7) | 43 (14.7) | <0.001 |
| Diabetic, n (%) | 138 (24.5) | 209 (33.1) | 0.191 |  | 91 (31.2) | 91 (31.2) | <0.001 |
| Liver diseases, n (%) | 125 (22.2) | 104 (16.5) | 0.145 |  | 61 (20.9) | 60 (20.5) | 0.008 |
| **Scoring systems** |  |  |  |  |  |  |  |
| CCI | 3.98 (3.04) | 5.56 (2.62) | 0.555 |  | 4.91 (3.10) | 4.99 (2.81) | 0.028 |
| APSIII | 64.13 (32.97) | 57.11 (28.15) | 0.229 |  | 59.16 (28.52) | 59.12 (29.00) | 0.001 |
| SAPSII | 39.83 (18.14) | 39.30 (15.04) | 0.032 |  | 39.35 (17.14) | 40.12 (15.57) | 0.047 |
| SOFA score | 8.20 (4.72) | 6.81 (4.19) | 0.313 |  | 7.46 (4.33) | 7.41 (4.34) | 0.011 |
| **Infection site, n (%)** |  |  |  |  |  |  |  |
| Bacteremia, n (%) | 21 (3.7) | 13 (2.1) | 0.1 |  | 12 (4.1) | 10 (3.4) | 0.036 |
| Abdominal infection, n (%) | 7 (1.2) | 10 (1.6) | 0.029 |  | 4 (1.4) | 6 (2.1) | 0.053 |
| Pneumonia, n (%) | 154 (27.4) | 190 (30.1) | 0.061 |  | 89 (30.5) | 96 (32.9) | 0.052 |
| SSTI, n (%) | 10 (1.8) | 12 (1.9) | 0.009 |  | 6 (2.1) | 5 (1.7) | 0.025 |
| UI, n (%) | 87 (15.5) | 113 (17.9) | 0.066 |  | 55 (18.8) | 57 (19.5) | 0.017 |
| **Interventions** |  |  |  |  |  |  |  |
| VIS | 12.61 (23.83) | 8.04 (23.94) | 0.191 |  | 9.27 (16.69) | 9.43 (32.26) | 0.006 |
| CRRT, n (%) | 83 (14.7) | 60 (9.5) | 0.161 |  | 27 (9.2) | 34 (11.6) | 0.078 |
| MV, n (%) | 359 (63.8) | 331 (52.5) | 0.231 |  | 174 (59.6) | 178 (61.0) | 0.028 |
| MV time, d | 1.99 (2.93) | 2.16 (2.99) | 0.058 |  | 1.89 (3.19) | 2.29 (3.13) | 0.128 |
| Mannitol, n (%) | 18 (3.2) | 16 (2.5) | 0.04 |  | 9 (3.1) | 10 (3.4) | 0.019 |
| Sodium bicarbonate, n (%) | 76 (13.5) | 61 (9.7) | 0.12 |  | 30 (10.3) | 30 (10.3) | <0.001 |
| Statin, n (%) | 65 (11.5) | 228 (36.1) | 0.603 |  | 56 (19.2) | 55 (18.8) | 0.009 |
| Calcium supplementation, n (%) | 293 (52.0) | 303 (48.0) | 0.081 |  | 154 (52.7) | 149 (51.0) | 0.034 |
| Magnesium Sulfate, n (%) | 71 (12.6) | 121 (19.2) | 0.18 |  | 40 (13.7) | 44 (15.1) | 0.039 |
| Potassium chloride, n (%) | 424 (75.3) | 504 (79.9) | 0.11 |  | 226 (77.4) | 232 (79.5) | 0.05 |
| DC Cardioversion, n (%) | 9 (1.6) | 28 (4.4) | 0.167 |  | 6 (2.1) | 4 (1.4) | 0.053 |

Abbreviations: SD standard deviation; SMD standardized mean difference; SOFA Sequential Organ Failure Assessment; CCI charlson comorbidity index; SpO_2_ Peripheral capillary oxygen saturation; SBP systolic blood pressure; DBP Diastolic blood pressure; MBP mean arterial pressure; APSIII Acute Physiology Score III; SAPS II Simpliﬁed Acute Physiology Score II; SOFA Sequential Organ Failure Assessment; RBC red blood cell counts; WBC white blood cell counts; T-Bil Bilirubin total; BUN blood urine nitrogen; INR International normalized ratio; PT Prothrombin time; PTT Activated partial thromboplastin time; CRRT Continuous renal replacement therapy; MI Myocardial infarct; CHF Congestive heart failure; PVD Peripheral Vascular Disease; CVD Cerebra Vascular Disease; CPD Chronic pulmonary disease; RD renal disease；SSTI Skin and soft tissues infection; UI Urinary infection; MV mechanical ventilation; VIS The vasoactive-inotropic score was calculated as follows: dopamine dose (in micrograms per kilogram per minute) + dobutamine dose (in micrograms per kilogram per minute) + 100 × epinephrine dose (in micrograms per kilogram per minute) + 10 × milrinone dose (in micrograms per kilogram per minute) + 10000 × vasopressin dose (in international units per kilogram per minute) + 100 × norepinephrine dose (in micrograms per kilogram per minute).
