# Supplementary material for: Association between the use of β-adrenergic receptor blockers and all-cause mortality in sepsis-associated rhabdomyolysis syndrome: a cohort study
Source: Front Med (Lausanne). 2026 Feb 13;13:1743813. doi: 10.3389/fmed.2026.1743813 (PMC12946102; doi:10.3389/fmed.2026.1743813)
Supplement: Supplementary file 3 [file Table_3.docx]

**Supplementary Table 3. Baseline characteristics of β-blocker use in SAR**

| Variables | Before propensity score matching | | |  | After propensity score matching | | |
| --- | --- | --- | --- | --- | --- | --- | --- |
|  | Non-β - blockers  (n = 563) | β- blockers  (n = 631) | p |  | Non-β-blockers  (n = 292) | β- blockers  (n = 292) | p |
| Age, Median (IQR) | 54.8 (40.0, 67.1) | 65.2 (55.4, 76.2) | < 0.001 |  | 59.3 (47.9, 72.2) | 60.7 (48.9, 72.6) | 0.877 |
| Sex, n (%) |  |  | 0.165 |  |  |  | 0.599 |
| Female | 198 (35.2) | 198 (31.4) |  |  | 95 (32.5) | 101 (34.6) |  |
| Male | 365 (64.8) | 433 (68.6) |  |  | 197 (67.5) | 191 (65.4) |  |
| Race, n (%) |  |  | 0.002 |  |  |  | 0.832 |
| Africa American | 56 (9.9) | 40 (6.3) |  |  | 30 (10.3) | 31 (10.6) |  |
| White | 286 (50.8) | 379 (60.1) |  |  | 156 (53.4) | 162 (55.5) |  |
| Other | 221 (39.3) | 212 (33.6) |  |  | 106 (36.3) | 99 (33.9) |  |
| BMI (kg/m^2^), Median (IQR) | 27.2 (24.3, 32.0) | 28.0 (24.6, 32.5) | 0.212 |  | 27.7 (24.5, 32.4) | 27.7 (24.4, 33.0) | 0.718 |
| 28-day Mortality, n (%) | 177 (31.4) | 97 (15.4) | < 0.001 |  | 77 (26.4) | 51 (17.5) | 0.009 |
| 60-day Mortality, n (%) | 188 (33.4) | 116 (18.4) | < 0.001 |  | 86 (29.5) | 53 (18.2) | 0.001 |
| 90-day Mortality, n (%) | 195 (34.6) | 127 (20.1) | < 0.001 |  | 91 (31.2) | 56 (19.2) | < 0.001 |
| In-hospital mortality, n (%) | 172 (30.6) | 97 (15.4) | < 0.001 |  | 77 (26.4) | 49 (16.8) | 0.005 |
| ICU Mortality, n (%) | 156 (27.7) | 80 (12.7) | < 0.001 |  | 68 (23.3) | 40 (13.7) | 0.003 |
| Length of ICU stay(days),  Median (IQR) | 2.9 (1.9, 5.7) | 4.0 (2.3, 7.6) | < 0.001 |  | 3.2 (1.9, 6.2) | 4.0 (2.2, 7.8) | 0.007 |
| Length of hospital stay(days),  Median (IQR) | 6.7 (3.9, 11.7) | 8.2 (5.3, 14.5) | < 0.001 |  | 7.5 (4.8, 12.7) | 9.1 (5.7, 15.1) | 0.002 |
| ICU type, n (%) |  |  | < 0.001 |  |  |  | 0.921 |
| CCU | 71 (12.6) | 201 (31.9) |  |  | 52 (17.8) | 52 (17.8) |  |
| CVICU | 21 (3.7) | 86 (13.6) |  |  | 18 (6.2) | 21 (7.2) |  |
| MICU | 283 (50.3) | 152 (24.1) |  |  | 111 (38) | 104 (35.6) |  |
| SICU | 62 (11) | 70 (11.1) |  |  | 38 (13) | 44 (15.1) |  |
| Other | 126 (22.4) | 122 (19.3) |  |  | 73 (25) | 71 (24.3) |  |
| Year of Admission, n (%) |  |  | < 0.001 |  |  |  | 0.931 |
| 2008-2010 | 123 (21.8) | 211 (33.4) |  |  | 83 (28.4) | 76 (26) |  |
| 2011-2013 | 120 (21.3) | 170 (26.9) |  |  | 73 (25) | 76 (26) |  |
| 2014-2016 | 157 (27.9) | 139 (22) |  |  | 66 (22.6) | 69 (23.6) |  |
| 2017-2019 | 163 (29) | 111 (17.6) |  |  | 70 (24) | 71 (24.3) |  |
| **Vital signs** |  |  |  |  |  |  |  |
| Heart rate(/min), Median (IQR) | 108.0 (96.0, 120.8) | 105.0 (94.0, 119.0) | 0.194 |  | 109.0 (97.0, 121.2) | 106.0 (96.0, 119.0) | 0.267 |
| SBP (mm Hg), Median (IQR) | 88.0 (78.0, 101.0) | 87.0 (79.0, 97.8) | 0.782 |  | 89.0 (80.0, 101.0) | 89.5 (80.0, 100.0) | 0.958 |
| DBP (mm Hg), Median (IQR) | 47.0 (40.0, 54.0) | 47.0 (40.0, 54.0) | 0.776 |  | 48.0 (41.0, 55.0) | 48.0 (41.0, 55.0) | 0.685 |
| MBP (mm Hg), Median (IQR) | 59.0 (52.0, 66.0) | 60.0 (52.6, 66.0) | 0.487 |  | 60.0 (53.0, 68.0) | 61.0 (54.0, 67.1) | 0.832 |
| Resprate (/min), Median (IQR) | 29.0 (24.5, 33.0) | 27.0 (24.0, 31.0) | < 0.001 |  | 28.0 (24.0, 32.0) | 28.0 (24.4, 32.0) | 0.821 |
| Temperature (℃), Median (IQR) | 37.6 (37.1, 38.2) | 37.4 (37.1, 38.1) | 0.092 |  | 37.7 (37.2, 38.3) | 37.6 (37.1, 38.2) | 0.313 |
| SpO_2_(%), Median (IQR) | 92.0 (89.0, 95.0) | 92.5 (90.0, 95.0) | 0.227 |  | 93.0 (90.0, 95.0) | 93.0 (90.0, 95.0) | 0.984 |
| **Laboratory tests** |  |  |  |  |  |  |  |
| Hematocrit (%), Mean ± SD | 33.0 (28.4, 37.2) | 33.6 (28.4, 37.9) | 0.16 |  | 32.8 ± 6.2 | 32.9 ± 7.1 | 0.84 |
| Hemoglobin(g/dL), Mean ± SD | 11.0 (9.4, 12.4) | 11.4 (9.7, 12.9) | 0.004 |  | 11.0 ± 2.1 | 11.0 ± 2.4 | 0.892 |
| Platelets(10^9^/L), Median (IQR) | 158.5 (112.0, 214.0) | 170.0 (126.0, 222.0) | 0.034 |  | 165.0 (115.8, 219.2) | 165.0 (115.5, 220.2) | 0.999 |
| WBC (10^9^/L), Median (IQR) | 15.1 (11.1, 20.0) | 14.4 (11.0, 19.1) | 0.14 |  | 15.1 (10.7, 19.2) | 14.9 (11.4, 19.8) | 0.781 |
| Albumin (g/dL), Median (IQR) | 3.2 (2.8, 3.7) | 3.2 (2.8, 3.7) | 0.764 |  | 3.3 (2.9, 3.7) | 3.2 (2.8, 3.7) | 0.641 |
| Bicarbonate (mEq/L), Median (IQR) | 18.0 (15.0, 22.0) | 20.0 (17.0, 23.0) | < 0.001 |  | 19.0 (16.0, 22.0) | 19.5 (16.0, 23.0) | 0.677 |
| BUN (mg/dL), Median (IQR) | 26.0 (16.0, 43.0) | 24.0 (17.0, 36.0) | 0.063 |  | 25.0 (15.0, 40.0) | 24.0 (16.0, 39.0) | 0.959 |
| Creatinine (mg/dL), Median (IQR) | 1.6 (1.0, 2.9) | 1.3 (1.0, 2.0) | < 0.001 |  | 1.4 (0.9, 2.6) | 1.3 (1.0, 2.4) | 0.934 |
| Calcium (mg/dL), Median (IQR) | 7.6 (7.0, 8.1) | 7.9 (7.3, 8.5) | < 0.001 |  | 7.7 (7.1, 8.1) | 7.8 (7.1, 8.2) | 0.72 |
| Chloride (mg/dL), Median (IQR) | 107.0 (104.0, 111.0) | 106.0 (103.0, 110.0) | 0.004 |  | 107.0 (103.0, 111.0) | 107.0 (103.0, 111.0) | 0.933 |
| INR, Median (IQR) | 1.3 (1.2, 1.7) | 1.2 (1.1, 1.5) | < 0.001 |  | 1.3 (1.1, 1.6) | 1.3 (1.1, 1.5) | 0.325 |
| PT(s), Median (IQR) | 14.4 (12.8, 18.1) | 13.9 (12.5, 16.2) | 0.003 |  | 14.0 (12.5, 17.3) | 13.9 (12.5, 16.1) | 0.529 |
| PTT(s), Median (IQR) | 32.4 (27.7, 53.4) | 39.4 (29.4, 88.7) | < 0.001 |  | 32.8 (27.3, 56.0) | 33.2 (28.1, 57.2) | 0.608 |
| TBil (mg/dL), Median (IQR) | 0.8 (0.4, 1.4) | 0.7 (0.5, 1.2) | 0.363 |  | 0.8 (0.4, 1.3) | 0.7 (0.4, 1.2) | 0.861 |
| Creatine kinase (IU/L), Median (IQR) | 2785.0 (1563.5, 7104.0) | 2249.0 (1411.5, 4750.0) | < 0.001 |  | 2474.0 (1471.5, 6626.0) | 2366.0 (1494.5, 5300.8) | 0.546 |
| Lactate, Median (IQR) | 2.7 (1.5, 5.9) | 2.6 (1.6, 4.8) | 0.383 |  | 2.0 (1.3, 4.0) | 2.4 (1.6, 3.9) | 0.04 |
| pH, Median (IQR) | 7.3 (7.2, 7.3) | 7.3 (7.2, 7.4) | < 0.001 |  | 7.3 (7.2, 7.4) | 7.3 (7.2, 7.4) | 0.821 |
| PO2(mm Hg), Median (IQR) | 76.0 (59.0, 98.0) | 81.0 (66.0, 109.0) | < 0.001 |  | 82.0 (65.8, 112.0) | 79.0 (65.0, 104.0) | 0.426 |
| PCO2(mm Hg), Median (IQR) | 46.0 (39.0, 55.0) | 45.0 (39.0, 53.0) | 0.307 |  | 44.0 (37.0, 52.0) | 45.0 (39.0, 53.0) | 0.26 |
| Sodium (mmol/L), Median (IQR) | 139.0 (136.0, 142.0) | 138.0 (135.0, 141.0) | < 0.001 |  | 139.0 (136.0, 141.0) | 139.0 (135.8, 142.0) | 0.772 |
| Potassium(mmol/L), Median (IQR) | 4.2 (3.7, 4.8) | 4.2 (3.8, 4.6) | 0.847 |  | 4.2 (3.7, 4.7) | 4.1 (3.7, 4.6) | 0.873 |
| Phosphate(mmol/L), Median (IQR) | 3.8 (2.8, 5.1) | 3.6 (2.8, 4.5) | 0.005 |  | 3.7 (2.8, 4.9) | 3.6 (2.8, 4.8) | 0.777 |
| Magnesium(mmol/L), Median (IQR) | 2.0 (1.7, 2.3) | 2.0 (1.7, 2.2) | 0.064 |  | 2.0 (1.7, 2.2) | 2.0 (1.7, 2.2) | 0.691 |
| Glucose(mg/dL), Median (IQR) | 131.0 (104.0, 183.0) | 145.0 (114.0, 192.0) | < 0.001 |  | 134.0 (106.0, 189.2) | 141.0 (110.0, 195.5) | 0.273 |
| **Comorbidities, n (%)** |  |  |  |  |  |  |  |
| MI, n (%) | 120 (21.3) | 314 (49.8) | < 0.001 |  | 88 (30.1) | 90 (30.8) | 0.857 |
| CHF, n (%) | 90 (16) | 241 (38.2) | < 0.001 |  | 73 (25) | 80 (27.4) | 0.51 |
| PVD, n (%) | 30 (5.3) | 98 (15.5) | < 0.001 |  | 20 (6.8) | 20 (6.8) | 1 |
| CVD, n (%) | 82 (14.6) | 99 (15.7) | 0.589 |  | 57 (19.5) | 59 (20.2) | 0.836 |
| CPD, n (%) | 138 (24.5) | 138 (21.9) | 0.28 |  | 69 (23.6) | 69 (23.6) | 1 |
| RD, n (%) | 58 (10.3) | 123 (19.5) | < 0.001 |  | 43 (14.7) | 43 (14.7) | 1 |
| Diabetic, n (%) | 138 (24.5) | 209 (33.1) | 0.001 |  | 91 (31.2) | 91 (31.2) | 1 |
| Liver diseases, n (%) | 125 (22.2) | 104 (16.5) | 0.012 |  | 61 (20.9) | 60 (20.5) | 0.919 |
| CCI, Median (IQR) | 4.0 (1.0, 6.0) | 5.0 (4.0, 7.0) | < 0.001 |  | 5.0 (3.0, 7.0) | 5.0 (3.0, 7.0) | 0.567 |
| Severity score |  |  |  |  |  |  |  |
| APSIII, Median (IQR) | 56.0 (38.0, 84.0) | 49.0 (35.0, 75.5) | < 0.001 |  | 52.0 (38.0, 74.2) | 51.0 (37.0, 80.0) | 0.906 |
| SAPSII, Median (IQR) | 37.0 (26.0, 51.5) | 37.0 (28.0, 48.0) | 0.915 |  | 36.0 (26.8, 49.2) | 38.0 (29.0, 50.0) | 0.333 |
| SOFA score, Median (IQR) | 7.0 (4.0, 12.0) | 6.0 (3.0, 9.0) | < 0.001 |  | 6.0 (4.0, 10.2) | 6.0 (4.0, 10.0) | 0.924 |
| **Infection site, n (%)** |  |  |  |  |  |  |  |
| Bacteremia, n (%) | 21 (3.7) | 13 (2.1) | 0.083 |  | 12 (4.1) | 10 (3.4) | 0.664 |
| Abdominal infection, n (%) | 7 (1.2) | 10 (1.6) | 0.619 |  | 4 (1.4) | 6 (2.1) | 0.524 |
| Pneumonia, n (%) | 154 (27.4) | 190 (30.1) | 0.294 |  | 89 (30.5) | 96 (32.9) | 0.534 |
| SSTI, n (%) | 10 (1.8) | 12 (1.9) | 0.872 |  | 6 (2.1) | 5 (1.7) | 0.761 |
| UTI, n (%) | 87 (15.5) | 113 (17.9) | 0.257 |  | 55 (18.8) | 57 (19.5) | 0.834 |
| **Treatments** |  |  |  |  |  |  |  |
| VIP, Median (IQR) | 0.0 (0.0, 16.8) | 0.0 (0.0, 9.6) | 0.013 |  | 0.0 (0.0, 12.5) | 0.0 (0.0, 11.3) | 0.556 |
| CRRT, n (%) | 83 (14.7) | 60 (9.5) | 0.005 |  | 27 (9.2) | 34 (11.6) | 0.344 |
| MV, n (%) | 359 (63.8) | 331 (52.5) | < 0.001 |  | 174 (59.6) | 178 (61) | 0.735 |
| MV time, Median (IQR) | 1.1 (0.5, 2.4) | 1.2 (0.5, 2.6) | 0.459 |  | 1.0 (0.4, 2.2) | 1.2 (0.4, 2.8) | 0.123 |
| Mannitol, n (%) | 18 (3.2) | 16 (2.5) | 0.493 |  | 9 (3.1) | 10 (3.4) | 0.816 |
| Sodium bicarbonate, n (%) | 76 (13.5) | 61 (9.7) | 0.038 |  | 30 (10.3) | 30 (10.3) | 1 |
| Statin, n (%) | 65 (11.5) | 228 (36.1) | < 0.001 |  | 56 (19.2) | 55 (18.8) | 0.916 |
| Calcium supplementation, n (%) | 293 (52) | 303 (48) | 0.165 |  | 154 (52.7) | 149 (51) | 0.679 |
| Magnesium Sulfate, n (%) | 71 (12.6) | 121 (19.2) | 0.002 |  | 40 (13.7) | 44 (15.1) | 0.637 |
| Potassium chloride, n (%) | 424 (75.3) | 504 (79.9) | 0.059 |  | 226 (77.4) | 232 (79.5) | 0.546 |
| DC Cardioversion, n (%) | 9 (1.6) | 28 (4.4) | 0.005 |  | 6 (2.1) | 4 (1.4) | 0.524 |

Abbreviations: SD, standard deviation; SMD, standardized mean difference; SOFA, Sequential Organ Failure Assessment; WBC, white blood cell.

T: Temperature; HR heart rate; RR Respiratory rate; CCI Charlson comorbidity index; SpO2 Peripheral capillary oxygen saturation; SBP systolic blood pressure; DBP Diastolic blood pressure; MBP mean arterial pressure; APSIII Acute Physiology Score III; SAPS II Simpliﬁed Acute Physiology Score II; SOFA Sequential Organ Failure Assessment; RBC red blood cell counts; WBC white blood cell counts; T-Bil Bilirubin total; BUN blood urine nitrogen; INR International normalized ratio; PT Prothrombin time; PTT Activated partial thromboplastin time; CRRT Continuous renal replacement therapy; MI Myocardial infarct; CHF Congestive heart failure; PVD Peripheral Vascular Disease; CVD Cerebro Vascular Disease; CPD Chronic pulmonary disease; RD renal disease；SSTI Skin and soft tissues infection; UI Urinary infection; MV mechanical ventilation; VIS The vasoactive-inotropic score was calculated as follows: dopamine dose (in micrograms per kilogram per minute) + dobutamine dose (in micrograms per kilogram per minute) + 100 × epinephrine dose (in micrograms per kilogram per minute) + 10 × milrinone dose (in micrograms per kilogram per minute) + 10000 × vasopressin dose (in international units per kilogram per minute) + 100 × norepinephrine dose (in micrograms per kilogram per minute).
