# Supplementary material for: Association between the use of β-adrenergic receptor blockers and all-cause mortality in sepsis-associated rhabdomyolysis syndrome: a cohort study
Source: Front Med (Lausanne). 2026 Feb 13;13:1743813. doi: 10.3389/fmed.2026.1743813 (PMC12946102; doi:10.3389/fmed.2026.1743813)
Supplement: Supplementary file 4 [file Table_4.docx]

**Supplementary Table 4. Baseline characteristics of in-hospital mortality in the Entire cohort and the matched cohort**

| Variables | Total  (n = 1194) | Entire Cohort | | p | Total  (n = 584) | Matched Cohort | | p |
| --- | --- | --- | --- | --- | --- | --- | --- | --- |
|  |  | Survivors  (n = 925) | Non-Survivors  (n = 269) |  |  | Survivors  (n = 458) | Non-Survivors  (n = 126) |  |
| **β-blocker** |  |  |  |  |  |  |  |  |
| Metoprolol, n (%) | 590 (49.4) | 500 (54.1) | 90 (33.5) | < 0.001 | 265 (45.4) | 223 (48.7) | 42 (33.3) | 0.002 |
| Labetalol, n (%) | 168 (14.1) | 127 (13.7) | 41 (15.2) | 0.53 | 98 (16.8) | 77 (16.8) | 21 (16.7) | 0.969 |
| Atenolol, n (%) | 30 ( 2.5) | 30 (3.2) | 0 (0) | 0.003 | 19 ( 3.3) | 19 (4.1) | 0 (0) | 0.019 |
| Betaxolol, n (%) | 30 ( 2.5) | 30 (3.2) | 0 (0) | 0.003 | 19 ( 3.3) | 19 (4.1) | 0 (0) | 0.019 |
| Carvedilol, n (%) | 31 ( 2.6) | 27 (2.9) | 4 (1.5) | 0.194 | 13 ( 2.2) | 12 (2.6) | 1 (0.8) | 0.317 |
| Pindolol, n (%) | 1 ( 0.1) | 1 (0.1) | 0 (0) | 1 | 1 ( 0.2) | 1 (0.2) | 0 (0) | 1 |
| Propranolol, n (%) | 10 ( 0.8) | 8 (0.9) | 2 (0.7) | 1 | 6 ( 1.0) | 5 (1.1) | 1 (0.8) | 1 |
| Nadolol, n (%) | 6 ( 0.5) | 3 (0.3) | 3 (1.1) | 0.132 | 4 ( 0.7) | 1 (0.2) | 3 (2.4) | 0.033 |
| Esmolol, n (%) | 28 ( 2.3) | 16 (1.7) | 12 (4.5) | 0.009 | 14 ( 2.4) | 6 (1.3) | 8 (6.3) | 0.004 |
| Acebutolol, n (%) | 1 ( 0.1) | 1 (0.1) | 0 (0) | 1 | 1 ( 0.2) | 1 (0.2) | 0 (0) | 1 |
| First time after intime, (hours) | 2.5 (0.0, 35.6) | 4.1 (0.0, 43.7) | 0.0 (0.0, 10.5) | < 0.001 | 2.3 (0.0, 42.9) | 4.4 (0.0, 49.6) | 0.0 (0.0, 16.7) | < 0.001 |
| Total duration of medication, (hours) | 27.5 (0.0, 145.8) | 48.0 (0.0, 168.0) | 0.0 (0.0, 49.0) | < 0.001 | 23.0 (0.0, 144.0) | 38.0 (0.0, 167.2) | 0.0 (0.0, 48.8) | < 0.001 |
| **Route of administration, n (%)** | |  |  | < 0.001 |  |  |  | < 0.001 |
| No | 510 (42.7) | 354 (38.3) | 156 (58) |  | 261 (44.7) | 191 (41.7) | 70 (55.6) |  |
| IV | 93 ( 7.8) | 42 (4.5) | 51 (19) |  | 50 ( 8.6) | 28 (6.1) | 22 (17.5) |  |
| PO/NG | 283 (23.7) | 267 (28.9) | 16 (5.9) |  | 129 (22.1) | 118 (25.8) | 11 (8.7) |  |
| IV and PO/NG | 308 (25.8) | 262 (28.3) | 46 (17.1) |  | 144 (24.7) | 121 (26.4) | 23 (18.3) |  |
| **Infection site, n(%)** |  |  |  |  |  |  |  |  |
| Bacteremia, n (%) | 35 ( 2.9) | 33 (3.6) | 2 (0.7) | 0.016 | 23 ( 3.9) | 23 (5) | 0 (0) | 0.007 |
| Abdominal infection, n (%) | 18 ( 1.5) | 11 (1.2) | 7 (2.6) | 0.149 | 11 ( 1.9) | 6 (1.3) | 5 (4) | 0.065 |
| Pneumonia, n (%) | 344 (28.8) | 258 (27.9) | 86 (32) | 0.194 | 185 (31.7) | 140 (30.6) | 45 (35.7) | 0.271 |
| Skin and tissue infection, n (%) | 22 ( 1.8) | 20 (2.2) | 2 (0.7) | 0.195 | 11 ( 1.9) | 11 (2.4) | 0 (0) | 0.133 |
| Urinary tract infection, n (%) | 200 (16.8) | 164 (17.7) | 36 (13.4) | 0.093 | 112 (19.2) | 98 (21.4) | 14 (11.1) | 0.009 |
| avg HR change, (IQR) | -2.5 (-6.4, 0.9) | -2.3 (-6.2, 0.8) | -3.8 (-7.3, 1.1) | 0.21 | -2.4 (-6.6, 0.8) | -2.5 (-6.5, 0.7) | -1.3 (-6.7, 1.2) | 0.704 |
| avg SBP change, (IQR) | -4.0 (-10.5, 2.1) | -4.0 (-10.4, 2.5) | -4.0 (-11.5, 1.2) | 0.66 | -4.6 (-13.9, 1.9) | -4.2 (-14.0, 2.8) | -5.4 (-13.6, 0.4) | 0.564 |
| avg DBP change, (IQR) | -1.5 (-5.5, 2.0) | -1.4 (-5.7, 2.0) | -1.5 (-5.0, 1.4) | 0.684 | -1.6 (-6.9, 2.0) | -1.5 (-7.1, 2.0) | -1.8 (-5.2, 0.9) | 0.8 |
| avg MBP change, (IQR) | 0.0 (-2.1, 0.0) | 0.0 (-2.5, 0.0) | 0.0 (-0.9, 0.0) | 0.588 | 0.0 (-1.9, 0.0) | 0.0 (-2.1, 0.0) | 0.0 (-1.8, 0.0) | 0.643 |
| avg HR-pre, Mean ± SD | 92.3 ± 15.6 | 91.1 ± 15.3 | 97.4 ± 16.2 | < 0.001 | 92.8 ± 15.9 | 92.1 ± 15.9 | 95.5 ± 15.9 | 0.162 |
| avg HR-post, (IQR) | 89.5 (79.2, 98.3) | 88.8 (78.2, 97.5) | 92.9 (82.0, 104.6) | 0.001 | 89.8 ± 15.2 | 89.0 ± 15.2 | 92.9 ± 15.0 | 0.08 |
| avg SBP-pre, (IQR) | 128.5 (114.1, 145.7) | 129.1 (114.8, 145.5) | 126.3 (112.6, 146.5) | 0.457 | 135.2 ± 20.9 | 135.8 ± 21.1 | 132.9 ± 20.3 | 0.36 |
| avg SBP-post, (IQR) | 124.4 (111.5, 138.5) | 125.3 (112.0, 139.0) | 119.6 (109.8, 134.5) | 0.124 | 128.8 ± 19.6 | 129.5 ± 19.8 | 125.6 ± 18.5 | 0.182 |
| avg DBP-pre, (IQR) | 69.8 (61.4, 78.1) | 70.3 (62.1, 78.0) | 66.5 (57.8, 79.6) | 0.168 | 72.1 ± 12.9 | 72.5 ± 12.6 | 70.6 ± 14.0 | 0.348 |
| avg DBP-post, (IQR) | 66.5 (60.0, 75.3) | 66.8 (61.0, 75.9) | 65.8 (57.4, 74.8) | 0.125 | 68.2 (61.3, 79.0) | 68.2 (61.6, 79.0) | 67.1 (58.8, 75.9) | 0.326 |
| avg MBP-pre, (IQR) | 0.0 (0.0, 89.3) | 69.3 (0.0, 90.5) | 0.0 (0.0, 82.1) | < 0.001 | 0.0 (0.0, 91.6) | 0.0 (0.0, 92.9) | 0.0 (0.0, 87.2) | 0.207 |
| avg MBP-post, (IQR) | 0.0 (0.0, 85.3) | 66.5 (0.0, 86.6) | 0.0 (0.0, 79.3) | < 0.001 | 0.0 (0.0, 86.8) | 0.0 (0.0, 87.7) | 0.0 (0.0, 83.0) | 0.278 |

Abbreviations: SBP systolic blood pressure; DBP Diastolic blood pressure; MBP mean arterial pressure; PO Oral administration; IV Intravenous injection; NG Nasogastric tube
