# Supplementary material for: Association between the use of β-adrenergic receptor blockers and all-cause mortality in sepsis-associated rhabdomyolysis syndrome: a cohort study
Source: Front Med (Lausanne). 2026 Feb 13;13:1743813. doi: 10.3389/fmed.2026.1743813 (PMC12946102; doi:10.3389/fmed.2026.1743813)
Supplement: Supplementary file 5 [file Table_5.docx]

**Supplementary Table 5. Univariate logistic analysis of the association between β -blocker use and in-hospital mortality**

| Variable | OR (95%CI) | P_value |
| --- | --- | --- |
| Sex (Male) | 1.06 (0.7~1.61) | 0.784 |
| Age | 1.03 (1.02~1.04) | <0.001 |
| Race (White) | 0.91 (0.46~1.82) | 0.792 |
| Race (Other) | 1.53 (0.76~3.1) | 0.232 |
| BMI | 0.97 (0.94~1) | 0.034 |
| Los ICU | 1.02 (0.99~1.05) | 0.193 |
| Los Hospital | 0.91 (0.88~0.95) | <0.001 |
| ICU type (CVICU) | 0.73 (0.34~1.57) | 0.426 |
| ICU type (MICU) | 0.26 (0.16~0.45) | <0.001 |
| ICU type (SICU) | 0.2 (0.1~0.43) | <0.001 |
| ICU type (Other ICU) | 0.21 (0.11~0.39) | <0.001 |
| Year of Admission (2011-2013) | 0.96 (0.54~1.69) | 0.875 |
| Year of Admission (2014-2016) | 1.08 (0.61~1.91) | 0.791 |
| Year of Admission (2017-2019) | 1.58 (0.92~2.71) | 0.097 |
| Heart rate | 1.01 (1~1.02) | 0.114 |
| SBP | 0.98 (0.97~0.99) | <0.001 |
| DBP | 0.98 (0.96~1) | 0.011 |
| MBP | 0.98 (0.97~1) | 0.019 |
| Respiratory rate | 1.01 (0.98~1.04) | 0.478 |
| Temperature | 0.66 (0.55~0.78) | <0.001 |
| SpO2 | 0.97 (0.95~0.99) | 0.006 |
| Hematocrit | 1 (0.97~1.03) | 0.993 |
| Hemoglobin | 0.97 (0.89~1.05) | 0.436 |
| Platelets | 1 (1~1) | 0.095 |
| WBC | 1.04 (1.02~1.07) | <0.001 |
| Albumin | 0.55 (0.4~0.74) | <0.001 |
| Bicarbonate | 0.86 (0.83~0.9) | <0.001 |
| BUN | 1.01 (1~1.02) | 0.011 |
| Creatinine | 1.05 (0.97~1.13) | 0.275 |
| Calcium | 0.91 (0.73~1.14) | 0.422 |
| Chloride | 1.01 (0.99~1.04) | 0.335 |
| INR | 1.38 (1.11~1.73) | 0.005 |
| PT | 1.04 (1.01~1.06) | 0.002 |
| PTT | 1.01 (1.01~1.02) | <0.001 |
| Bilirubin Total | 1.17 (1.05~1.3) | 0.005 |
| CK | 1 (1~1) | 0.112 |
| Lactate | 1.28 (1.2~1.37) | <0.001 |
| pH | 0.02 (0~0.09) | <0.001 |
| PO2 | 1 (1~1) | 0.731 |
| PCO2 | 1 (0.98~1.01) | 0.869 |
| Sodium | 0.98 (0.95~1.01) | 0.171 |
| Potassium | 1.18 (0.97~1.45) | 0.101 |
| Phosphate | 1.2 (1.08~1.32) | <0.001 |
| Magnesium | 1.96 (1.28~2.99) | 0.002 |
| Glucose | 1 (1~1.01) | <0.001 |
| Myocardial Infarct | 2.51 (1.67~3.77) | <0.001 |
| Congestive Heart Failure | 1.89 (1.24~2.88) | 0.003 |
| Peripheral Vascular Disease | 2.08 (1.05~4.11) | 0.036 |
| Cerebro Vascular Disease | 2.46 (1.57~3.85) | <0.001 |
| Chronic Pulmonary Disease | 0.67 (0.4~1.1) | 0.11 |
| Renal Disease | 1.72 (1.04~2.87) | 0.036 |
| Diabetic | 1.49 (0.99~2.25) | 0.059 |
| Liver diseases | 1.66 (1.06~2.62) | 0.028 |
| Charlson comorbidity index | 1.2 (1.12~1.28) | <0.001 |
| APSIII | 1.04 (1.03~1.04) | <0.001 |
| SAPSII | 1.06 (1.05~1.08) | <0.001 |
| SOFA score | 1.22 (1.16~1.28) | <0.001 |
| Bacteremia | 0 (0~Inf) | 0.976 |
| Abdominal infection | 3.74 (1.07~13.14) | 0.039 |
| Pneumonia | 1.26 (0.83~1.91) | 0.272 |
| Skin and Soft Tissue Infection | 0 (0~Inf) | 0.983 |
| Urinary tract infection | 0.46 (0.25~0.84) | 0.011 |
| VIP | 1.07 (1.06~1.09) | <0.001 |
| CRRT | 3.68 (2.12~6.37) | <0.001 |
| MV | 5.26 (3.09~8.96) | <0.001 |
| MV time | 1.09 (1.03~1.16) | 0.003 |
| β -blockers | 0.56 (0.38~0.84) | 0.005 |
| Mannitol | 2.19 (0.84~5.67) | 0.108 |
| Sodium bicarbonate | 5.23 (3~9.1) | <0.001 |
| Statin | 1.83 (1.15~2.91) | 0.011 |
| Calcium | 1.91 (1.27~2.88) | 0.002 |
| Magnesium Sulfate | 2.49 (1.52~4.1) | <0.001 |
| Potassium chloride | 1.01 (0.63~1.63) | 0.964 |
| DC Cardioversion | 8.92 (2.27~35.02) | 0.002 |
