# Supplementary material for: Association between the use of β-adrenergic receptor blockers and all-cause mortality in sepsis-associated rhabdomyolysis syndrome: a cohort study
Source: Front Med (Lausanne). 2026 Feb 13;13:1743813. doi: 10.3389/fmed.2026.1743813 (PMC12946102; doi:10.3389/fmed.2026.1743813)
Supplement: Supplementary file 6 [file Table_6.docx]

**Supplement Table 6. Sensitive analysis of the association between β-blocker use and mortality without trauma, intoxication and seizures**

| **Variable** | **n.total** | **n.event_%** | **Model 1** | |  | **Model 2** | |  | **Model3** | |
| --- | --- | --- | --- | --- | --- | --- | --- | --- | --- | --- |
|  |  |  | **HR (95%CI)** | **P value** |  | **HR (95%CI)** | **P value** |  | **HR (95%CI)** | **P value** |
| **In-hospital mortality** | |  |  |  |  |  |  |  |  |  |
| No β-blockers | 428 | 137 (32) | 1(Ref) |  |  | 1(Ref) |  |  | 1(Ref) |  |
| β-blockers | 508 | 75 (14.8) | 0.4 (0.3~0.53) | <0.001 |  | 0.28 (0.2~0.39) | <0.001 |  | 0.28 (0.2~0.39) | <0.001 |
|  |  |  |  |  |  |  |  |  |  |  |
| **ICU mortality** | |  |  |  |  |  |  |  |  |  |
| No β-blockers | 428 | 125 (29.2) | 1(Ref) |  |  | 1(Ref) |  |  | 1(Ref) |  |
| β-blockers | 508 | 61 (12) | 0.36 (0.26~0.49) | <0.001 |  | 0.29 (0.21~0.4) | <0.001 |  | 0.26 (0.18~0.38) | <0.001 |
|  |  |  |  |  |  |  |  |  |  |  |
| **28-day mortality** | |  |  |  |  |  |  |  |  |  |
| No β-blockers | 428 | 142 (33.2) | 1(Ref) |  |  | 1(Ref) |  |  | 1(Ref) |  |
| β-blockers | 508 | 75 (14.8) | 0.38 (0.29~0.51) | <0.001 |  | 0.29 (0.22~0.39) | <0.001 |  | 0.26 (0.18~0.36) | <0.001 |
|  |  |  |  |  |  |  |  |  |  |  |
| **90-day mortality** | |  |  |  |  |  |  |  |  |  |
| No β-blockers | 428 | 157 (36.7) | 1(Ref) |  |  | 1(Ref) |  |  | 1(Ref) |  |
| β-blockers | 508 | 100 (19.7) | 0.46 (0.35~0.59) | <0.001 |  | 0.35 (0.27~0.45) | <0.001 |  | 0.29 (0.21~0.39) | <0.001 |

Model 1: non adjusted

Model 2: sex, age, racce

Model 3: sex, age, race, ICU type, heart rate, temperature, peripheral capillary oxygen saturation, activated partial thromboplastin time, Renal Disease, Diabetic, mechanical ventilation, Mannitol, Statin
