# Supplementary material for: Association between the use of β-adrenergic receptor blockers and all-cause mortality in sepsis-associated rhabdomyolysis syndrome: a cohort study
Source: Front Med (Lausanne). 2026 Feb 13;13:1743813. doi: 10.3389/fmed.2026.1743813 (PMC12946102; doi:10.3389/fmed.2026.1743813)
Supplement: Supplementary file 7 [file Table_7.docx]

**Supplement Table 7. Association between β-blocker use and mortality in patients without heart failure and myocardial infarction**

| Variable | Event (%) | Followup.Time | Crude | |  | Adjusted | |
| --- | --- | --- | --- | --- | --- | --- | --- |
|  |  |  | HR (95%CI) | P value |  | HR (95%CI) | P value |
| **In-hospital mortality** |  |  |  |  |  |  |  |
| No β-blockers | 66 (19.5) | 100769.2758 | 1(Ref) |  |  | 1(Ref) |  |
| β-blockers | 17 (9.8) | 57453.67904 | 0.47 (0.27~0.8) | 0.005 |  | 0.43 (0.24~0.79) | 0.006 |
|  |  |  |  |  |  |  |  |
| **ICU mortality** |  |  |  |  |  |  |  |
| No β-blockers | 60 (17.8) | 100769.2758 | 1(Ref) |  |  | 1(Ref) |  |
| β-blockers | 15 (8.7) | 57453.67904 | 0.46 (0.26~0.8) | 0.007 |  | 0.41 (0.22~0.75) | 0.004 |
|  |  |  |  |  |  |  |  |
| **28-day mortality** |  |  |  |  |  |  |  |
| No β-blockers | 70 (20.7) | 100769.2758 | 1(Ref) |  |  | 1(Ref) |  |
| β-blockers | 16 (9.2) | 57453.67904 | 0.41 (0.24~0.71) | 0.001 |  | 0.3 (0.17~0.55) | <0.001 |
|  |  |  |  |  |  |  |  |
| **90-day mortality** |  |  |  |  |  |  |  |
| No β-blockers | 79 (23.4) | 100769.2758 | 1(Ref) |  |  | 1(Ref) |  |
| β-blockers | 21 (12.1) | 57453.67904 | 0.48 (0.3~0.77) | 0.003 |  | 0.36 (0.21~0.61) | <0.001 |

Crude: no adjusted

Adjusted for sex, age, race, ICUtype, heart rate, temperature, Mannitol, Statin, Diabetic, mechanical ventilation, activated partial thromboplastin time, Renal Disease, peripheral capillary oxygen saturation
