# Supplementary material for: Association between the use of β-adrenergic receptor blockers and all-cause mortality in sepsis-associated rhabdomyolysis syndrome: a cohort study
Source: Front Med (Lausanne). 2026 Feb 13;13:1743813. doi: 10.3389/fmed.2026.1743813 (PMC12946102; doi:10.3389/fmed.2026.1743813)
Supplement: Supplementary file 8 [file Table_8.docx]

**Supplementary Table 8. Subgroup analysis between β-blocker and in-hospital mortality**

| **Subgroup** | **Entire cohort** | | | |  | **Matched cohort** | | | |
| --- | --- | --- | --- | --- | --- | --- | --- | --- | --- |
|  | **Total** | **Event (%)** | **HR (95%CI)** | **P for interaction** |  | **Total** | **Event (%)** | **HR (95%CI)** | **P for interaction** |
| Overall |  |  | 0.31 (0.24~0.4) |  |  |  |  | 0.51 (0.36~0.75) |  |
| Sex |  |  |  | 0.014 |  |  |  |  | 0.408 |
| Female | 198 | 39 (19.7) | 0.43 (0.27~0.68) |  |  | 101 | 18 (17.8) | 0.87 (0.44~1.73) |  |
| Male | 433 | 58 (13.4) | 0.25 (0.18~0.36) |  |  | 191 | 31 (16.2) | 0.46 (0.29~0.72) |  |
| Race |  |  |  | 0.745 |  |  |  |  | 0.548 |
| Africa American | 40 | 6 (15) | 0.2 (0.06~0.65) |  |  | 31 | 5 (16.1) | 0.45 (0.08~2.34) |  |
| White | 379 | 47 (12.4) | 0.38 (0.25~0.58) |  |  | 162 | 19 (11.7) | 0.54 (0.3~0.97) |  |
| Other | 212 | 44 (20.8) | 0.33 (0.21~0.5) |  |  | 99 | 25 (25.3) | 0.68 (0.39~1.19) |  |
| Age |  |  |  | 0.465 |  |  |  |  | 0.479 |
| <65 | 315 | 43 (13.7) | 0.38 (0.24~0.58) |  |  | 182 | 26 (14.3) | 0.68 (0.39~1.21) |  |
| ≥65 | 316 | 54 (17.1) | 0.26 (0.18~0.38) |  |  | 110 | 23 (20.9) | 0.51 (0.3~0.87) |  |
| Sofa |  |  |  | <0.001 |  |  |  |  | 0.002 |
| <6 | 301 | 25 (8.3) | 0.78 (0.33~1.8) |  |  | 122 | 15 (12.3) | 1.42 (0.56~3.61) |  |
| ≥6 | 330 | 72 (21.8) | 0.24 (0.18~0.33) |  |  | 170 | 34 (20) | 0.37 (0.24~0.57) |  |
| Age and Sofa |  |  |  | 0.003 |  |  |  |  | 0.006 |
| Age<65 and Sofa <6 | 147 | 9 (6.1) | 8.97 (2.73~29.49) |  |  | 76 | 7 (9.2) | 5.83 (0.7~48.26) |  |
| Age<65 and Sofa ≥6 | 168 | 34 (20.2) | 0.28 (0.18~0.45) |  |  | 106 | 19 (17.9) | 0.44 (0.23~0.83) |  |
| Age≥65 and Sofa <6 | 154 | 16 (10.4) | 0.42 (0.16~1.11) |  |  | 46 | 8 (17.4) | 1.16 (0.38~3.48) |  |
| Age≥65 and Sofa ≥6 | 162 | 38 (23.5) | 0.22 (0.14~0.34) |  |  | 64 | 15 (23.4) | 0.37 (0.2~0.69) |  |
| CRRT |  |  |  | 0.27 |  |  |  |  | 0.012 |
| No | 571 | 76 (13.3) | 0.35 (0.25~0.48) |  |  | 258 | 41 (15.9) | 0.65 (0.43~0.98) |  |
| Yes | 60 | 21 (35) | 0.21 (0.12~0.39) |  |  | 34 | 8 (23.5) | 0.11 (0.04~0.34) |  |
| MV |  |  |  | 0.381 |  |  |  |  | 0.981 |
| No | 300 | 17 (5.7) | 0.33 (0.16~0.67) |  |  | 114 | 6 (5.3) | 0.64 (0.2~2.04) |  |
| Yes | 331 | 80 (24.2) | 0.29 (0.22~0.4) |  |  | 178 | 43 (24.2) | 0.5 (0.34~0.74) |  |
| Diabetic |  |  |  | 0.535 |  |  |  |  | 0.093 |
| No | 422 | 68 (16.1) | 0.32 (0.23~0.44) |  |  | 201 | 36 (17.9) | 0.64 (0.4~1.03) |  |
| Yes | 209 | 29 (13.9) | 0.24 (0.14~0.4) |  |  | 91 | 13 (14.3) | 0.34 (0.18~0.66) |  |
| Renal Disease |  |  |  | 0.415 |  |  |  |  | 0.298 |
| No | 508 | 80 (15.7) | 0.33 (0.24~0.45) |  |  | 249 | 42 (16.9) | 0.58 (0.39~0.88) |  |
| Yes | 123 | 17 (13.8) | 0.15 (0.08~0.3) |  |  | 43 | 7 (16.3) | 0.24 (0.09~0.66) |  |
| Chronic Pulmonary Disease |  |  |  | 0.431 |  |  |  |  | 0.801 |
| No | 493 | 79 (16) | 0.29 (0.21~0.4) |  |  | 223 | 41 (18.4) | 0.5 (0.33~0.76) |  |
| Yes | 138 | 18 (13) | 0.4 (0.22~0.73) |  |  | 69 | 8 (11.6) | 0.8 (0.31~2.1) |  |

Abbreviations: Sofa sequential organ failure assessment; CRRT continuous renal replacement therapy;MV mechanical ventilation
