# Supplementary material for: Association between the use of β-adrenergic receptor blockers and all-cause mortality in sepsis-associated rhabdomyolysis syndrome: a cohort study
Source: Front Med (Lausanne). 2026 Feb 13;13:1743813. doi: 10.3389/fmed.2026.1743813 (PMC12946102; doi:10.3389/fmed.2026.1743813)
Supplement: Supplementary file 9 [file Table_9.docx]

**Supplementary Table 9. Landmark analysis**

| Variable | landmark at 24h | | | |  | landmark at 48h | | | |
| --- | --- | --- | --- | --- | --- | --- | --- | --- | --- |
|  | n.total | n.event_% | OR(95%CI) | P_value |  | n.total | n.event_% | OR(95%CI) | P_value |
| Time below the landmark | | | | | | | | | |
| No β-blockers | 508 | 18 (3.5) | 1(Ref) |  |  | 508 | 62 (12.2) | 1(Ref) |  |
| β-blockers | 686 | 10 (1.5) | 0.407 (0.188~0.881) | 0.0226 |  | 686 | 22 (3.2) | 0.252 (0.155~0.409) | <0.001 |
| Time over the landmark | | | | | | | | | |
| No β-blockers | 490 | 138 (28.2) | 1(Ref) |  |  | 446 | 94 (21.1) | 1(Ref) |  |
| β-blockers | 676 | 103 (15.2) | 0.478 (0.37~0.617) | <0.001 |  | 664 | 91 (13.7) | 0.605 (0.453~0.807) | <0.001 |

Abbreviations: OR, odds ratio; CI, confidence interval
