# Supplementary material for: Association between the use of β-adrenergic receptor blockers and all-cause mortality in sepsis-associated rhabdomyolysis syndrome: a cohort study
Source: Front Med (Lausanne). 2026 Feb 13;13:1743813. doi: 10.3389/fmed.2026.1743813 (PMC12946102; doi:10.3389/fmed.2026.1743813)
Supplement: Supplementary file 10 [file Table_10.docx]

**Supplementary Table 10. Time‑dependent Cox proportional hazards model analysis**

| Variable | HR(95%CI) | P_value |  | p_value |
| --- | --- | --- | --- | --- |
| **β-blocker: strata t group** |  |  |  | 0.028 |
| No β-blocker: strata t group<24h | ref |  |  |  |
| β-blocker: strata t group<24h | 0.41 (0.19~0.902) | 0.0264 |  |  |
| No β-blocker: strata t group(24h, 48h) | ref |  |  |  |
| β-blocker: strata t group(24h, 48h) | 0.15 (0.074~0.297) | <0.001 |  |  |
| No β-blocker: strata t group(>=48h) | ref |  |  |  |
| β-blocker: strata t group(>=48h) | 0.39 (0.288~0.534) | <0.001 |  |  |
| **Sex** |  |  |  | 0.619 |
| Male | 0.98 (0.757~1.276) | 0.8949 |  |  |
| **Age** | 1.03 (1.018~1.035) | <0.001 |  | 0.008 |
| **ICU type** |  |  |  | 0.382 |
| CVICU | 0.57 (0.347~0.939) | 0.0272 |  |  |
| MICU | 0.65 (0.455~0.93) | 0.0184 |  |  |
| SICU | 0.56 (0.325~0.948) | 0.0312 |  |  |
| Other ICU | 0.62 (0.398~0.962) | 0.033 |  |  |
| **Calcium** | 0.89 (0.766~1.028) | 0.1105 |  | 0.789 |
| **PTT** | 1.01 (1.002~1.008) | 0.0014 |  | 0.967 |
| **Myocardial Infarct** | 0.93 (0.694~1.248) | 0.6301 |  | 0.686 |
| **Congestive Heart Failure** | 0.83 (0.619~1.117) | 0.22 |  | 0.023 |
| **VIS** | 1.01 (1.008~1.012) | <0.001 |  | 0.978 |
| **MV** | 4.45 (3.077~6.441) | <0.001 |  | 0.943 |

Abbreviations: HR hazard ratios; CI confidence interval

PTT Activated partial thromboplastin time; MV mechanical ventilation; VIS The vasoactive-inotropic score was calculated as follows: dopamine dose (in micrograms per kilogram per minute) + dobutamine dose (in micrograms per kilogram per minute) + 100 × epinephrine dose (in micrograms per kilogram per minute) + 10 × milrinone dose (in micrograms per kilogram per minute) + 10000 × vasopressin dose (in international units per kilogram per minute) + 100 × norepinephrine dose (in micrograms per kilogram per minute).
