# Supplementary material for: Association between the use of β-adrenergic receptor blockers and all-cause mortality in sepsis-associated rhabdomyolysis syndrome: a cohort study
Source: Front Med (Lausanne). 2026 Feb 13;13:1743813. doi: 10.3389/fmed.2026.1743813 (PMC12946102; doi:10.3389/fmed.2026.1743813)
Supplement: Supplementary file 11 [file Table_11.docx]

**Supplementary Table 11. Cox multivariate regression analysis of the association between β-blocker use and in-hospital mortality**

| **Variable** | **β-blocker used within 24 hours** | | | | | |  | **β-blocker used within 48 hours** | | | | | |
| --- | --- | --- | --- | --- | --- | --- | --- | --- | --- | --- | --- | --- | --- |
|  | **n.total** | **n.event_%** | **Unadjusted** | | **Adjusted** | |  | **n.total** | **n.event_%** | **Unadjusted** | | **Adjusted** | |
|  |  |  | **HR (95%CI)** | **P value** | **HR (95%CI)** | **P value** |  |  |  | **HR (95%CI)** | **P value** | **HR (95%CI)** | **P value** |
| **in-hospital mortality** | |  |  |  |  |  |  |  |  |  |  |  |  |
| No β-blockers | 508 | 178 (35) | 1(Ref) |  | 1(Ref) |  |  | 508 | 156 (30.7) | 1(Ref) |  | 1(Ref) |  |
| β-blockers | 338 | 80 (23.7) | 0.61 (0.47~0.79) | <0.001 | 0.52 (0.38~0.7) | <0.001 |  | 446 | 83 (18.6) | 0.54 (0.42~0.71) | <0.001 | 0.49 (0.36~0.67) | <0.001 |
|  |  |  |  |  |  |  |  |  |  |  |  |  |  |
| **ICU mortality** | |  |  |  |  |  |  |  |  |  |  |  |  |
| No β-blockers | 508 | 143 (28.1) | 1(Ref) |  | 1(Ref) |  |  | 508 | 143 (28.1) | 1(Ref) |  | 1(Ref) |  |
| β-blockers | 338 | 55 (16.3) | 0.53 (0.39~0.72) | <0.001 | 0.5 (0.35~0.72) | <0.001 |  | 446 | 68 (15.2) | 0.49 (0.37~0.65) | <0.001 | 0.45 (0.32~0.63) | <0.001 |
|  |  |  |  |  |  |  |  |  |  |  |  |  |  |
| **28-day mortality** | |  |  |  |  |  |  |  |  |  |  |  |  |
| No β-blockers | 508 | 160 (31.5) | 1(Ref) |  | 1(Ref) |  |  | 508 | 160 (31.5) | 1(Ref) |  | 1(Ref) |  |
| β-blockers | 338 | 65 (19.2) | 0.55 (0.41~0.74) | <0.001 | 0.49 (0.35~0.69) | <0.001 |  | 446 | 83 (18.6) | 0.53 (0.41~0.69) | <0.001 | 0.46 (0.34~0.63) | <0.001 |
|  |  |  |  |  |  |  |  |  |  |  |  |  |  |
| **90-day mortality** | |  |  |  |  |  |  |  |  |  |  |  |  |
| No β-blockers | 508 | 178 (35) | 1(Ref) |  | 1(Ref) |  |  | 508 | 178 (35) | 1(Ref) |  | 1(Ref) |  |
| β-blockers | 338 | 80 (23.7) | 0.61 (0.47~0.79) | <0.001 | 0.52 (0.38~0.7) | <0.001 |  | 446 | 102 (22.9) | 0.58 (0.45~0.74) | <0.001 | 0.49 (0.37~0.64) | <0.001 |

Abbreviations: HR hazard ratios; CI confidence interval

Entire cohort adjusted for sex, age, ICU type, calcium, activated partial thromboplastin time, Myocardial Infarct, Congestive Heart Failure, vasoactive-inotropic score, mechanical ventilation

Matched cohort Adjusted for sex, age, ICU type, temperature, activated partial thromboplastin time, Myocardial Infarct, vasoactive-inotropic score, Sodium bicarbonate
