# Supplementary material for: Association between the use of β-adrenergic receptor blockers and all-cause mortality in sepsis-associated rhabdomyolysis syndrome: a cohort study
Source: Front Med (Lausanne). 2026 Feb 13;13:1743813. doi: 10.3389/fmed.2026.1743813 (PMC12946102; doi:10.3389/fmed.2026.1743813)
Supplement: Supplementary file 12 [file Table_12.docx]

**Supplementary Figure legends**

**Supplementary Fig. 1：Percentage of missing data.**

BMI Body mass index; SpO_2_ Peripheral capillary oxygen saturation; SBP systolic blood pressure; DBP Diastolic blood pressure; MBP mean arterial pressure; WBC white blood cell counts; INR International normalized ratio; PT Prothrombin time; PTT Activated partial thromboplastin time

**Supplementary Fig. 2：ROC curves after propensity score matching (PSM)**

Receiver operating characteristic (ROC) curve for in-hospital mortality after propensity score matching (PSM)

**Supplementary Fig. 3：Absolute Standardized Mean Differences of Individual Covariates Before and After Propensity Score Matching**

SMD, standardized mean difference; BMI Body mass index; SOFA, Sequential Organ Failure Assessment; CCI charlson comorbidity index; SpO_2_ Peripheral capillary oxygen saturation; SBP systolic blood pressure; DBP Diastolic blood pressure; MBP mean arterial pressure; WBC white blood cell counts; T-Bil Bilirubin total; BUN blood urine nitrogen; INR International normalized ratio; PT Prothrombin time; PTT Activated partial thromboplastin time; CRRT Continuous renal replacement therapy; MV mechanical ventilation; VIS The vasoactive-inotropic score was calculated as follows: dopamine dose (in micrograms per kilogram per minute) + dobutamine dose (in micrograms per kilogram per minute) + 100 × epinephrine dose (in micrograms per kilogram per minute) + 10 × milrinone dose (in micrograms per kilogram per minute) + 10000 × vasopressin dose (in international units per kilogram per minute) + 100 × norepinephrine dose (in micrograms per kilogram per minute).

**Supplementary Fig. 4: Kaplan-Meier survival analysis curve and cumulative incidence of mortality in matched cohort.**

A. Kaplan-Meier survival analysis curve for β-blockers use and 28-day mortality

B. Kaplan-Meier survival analysis curve for β-blockers use and 90-day mortality

C. Kaplan-Meier survival analysis curve for β-blockers use and ICU mortality

**Supplementary Fig. 5: Kaplan-Meier survival analysis curve and cumulative incidence of mortality in entire cohort.**

A. Kaplan-Meier survival analysis curve for β-blockers use and in-hospital mortality

B. Kaplan-Meier survival analysis curve for β-blockers use and ICU mortality

C. Kaplan-Meier survival analysis curve for β-blockers use and 28 mortality

D. Kaplan-Meier survival analysis curve for β-blockers use and 90 mortality

**Supplementary Fig. 6: Landmark analysis evaluate the association between β‑blocker use and in-hospital mortality**

A. Landmark analysis evaluating the association between β‑blocker administration within the initial 24‑hour window and in-hospital mortality. Patients who died before the 24-hour landmark were excluded to minimize immortal time bias. Exposure status was defined based on β‑blocker use prior to the landmark time, and follow‑up commenced at the landmark point.

B. Landmark analysis assessing the association between β‑blocker administration within the first 48 hours and in-hospital mortality. Individuals who died before the 48-hour landmark were excluded. β-blocker exposure was classified according to treatment received before the landmark, and outcome risk was estimated starting from the 48-hour time point.

**Supplementary Fig. 7: Time‑dependent Cox proportional hazards model analysis**

β‑blocker exposure was treated as a time‑dependent covariate in the entire cohort and stratified into three temporal strata according to the timing of initiation (within 24 hours, 24–48 hours, and >48 hours). A time‑dependent Cox proportional hazards model was then applied to evaluate the association between β-blocker use and in-hospital mortality.
