# Supplementary material for: Association between the use of β-adrenergic receptor blockers and all-cause mortality in sepsis-associated rhabdomyolysis syndrome: a cohort study
Source: Front Med (Lausanne). 2026 Feb 13;13:1743813. doi: 10.3389/fmed.2026.1743813 (PMC12946102; doi:10.3389/fmed.2026.1743813)
Supplement: Supplementary file 13 [file Data_Sheet_1.pdf]

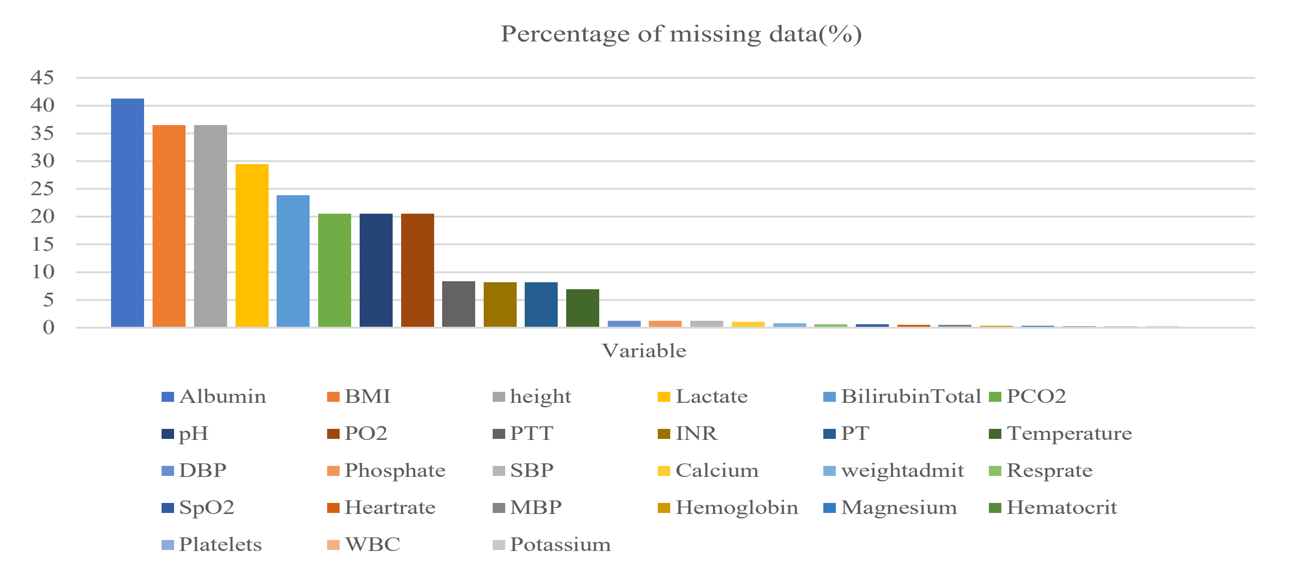

Percentage of missing data(%)
45
40
35
30
25
20
15
10
5
0
Variable
Albumin
BMI
height
Lactate
BilirubinTotal
PCO2
pH
PO2
PTT
INR
PT
Temperature
DBP
Phosphate
SBP
Calcium
weightadmit
Resprate
SpO2
Heartrate
MBP
Hemoglobin
Magnesium
Hematocrit
Platelets
WBC
Potassium
