# Supplementary figures and images for: Association between the use of β-adrenergic receptor blockers and all-cause mortality in sepsis-associated rhabdomyolysis syndrome: a cohort study
Source: Front Med (Lausanne). 2026 Feb 13;13:1743813. doi: 10.3389/fmed.2026.1743813 (PMC12946102; doi:10.3389/fmed.2026.1743813)

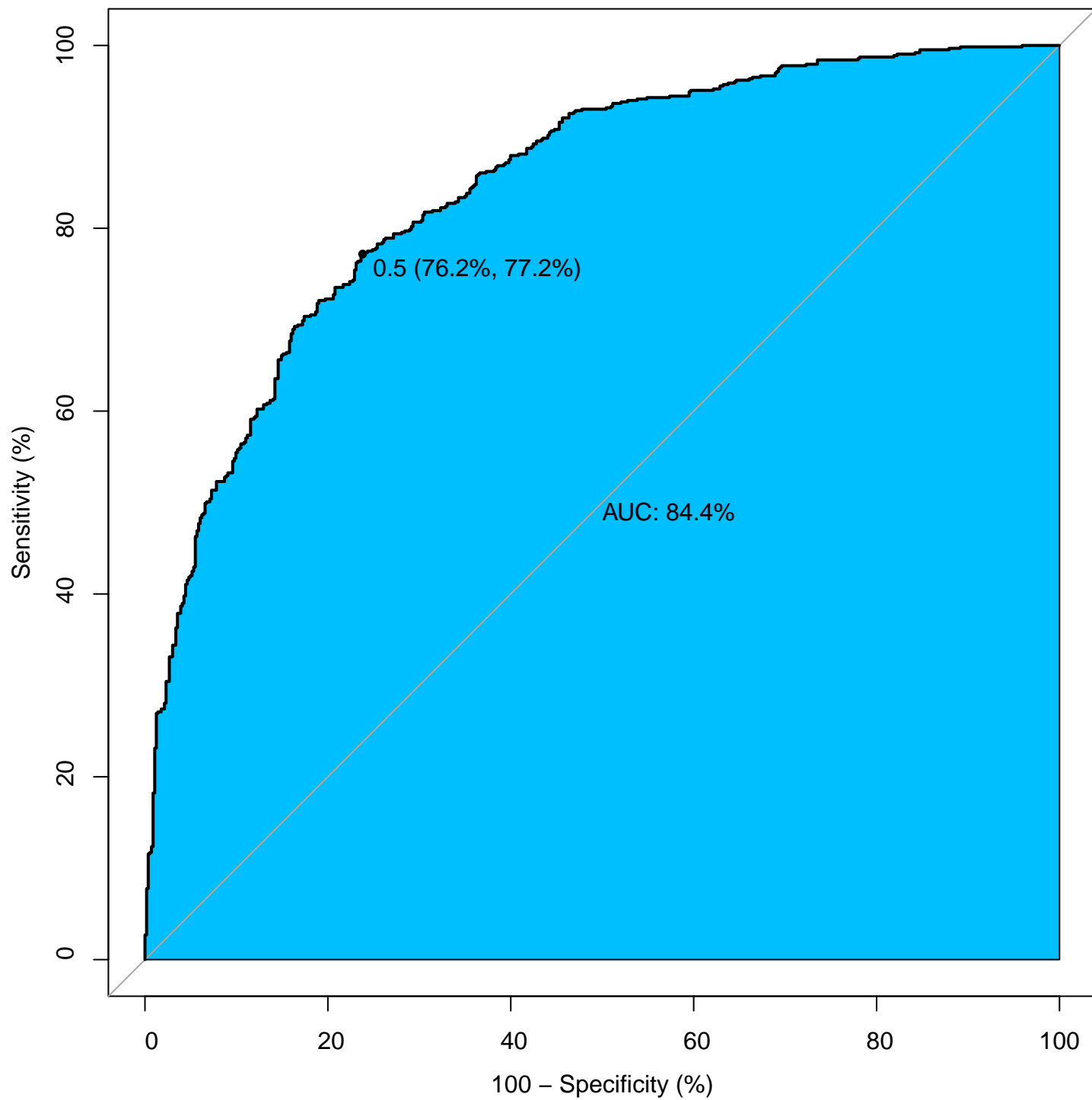

Supplement: Supplementary file 14 [file Data_Sheet_2.pdf]

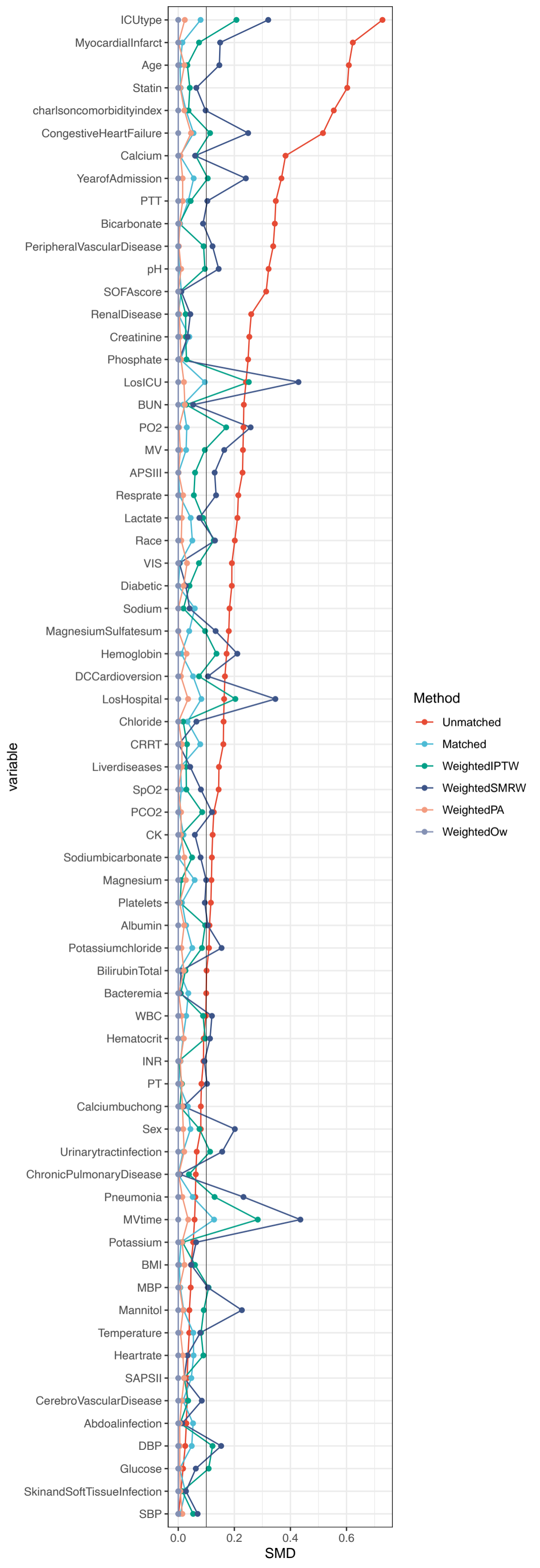

Supplement: Supplementary file 15 [file Data_Sheet_3.pdf]

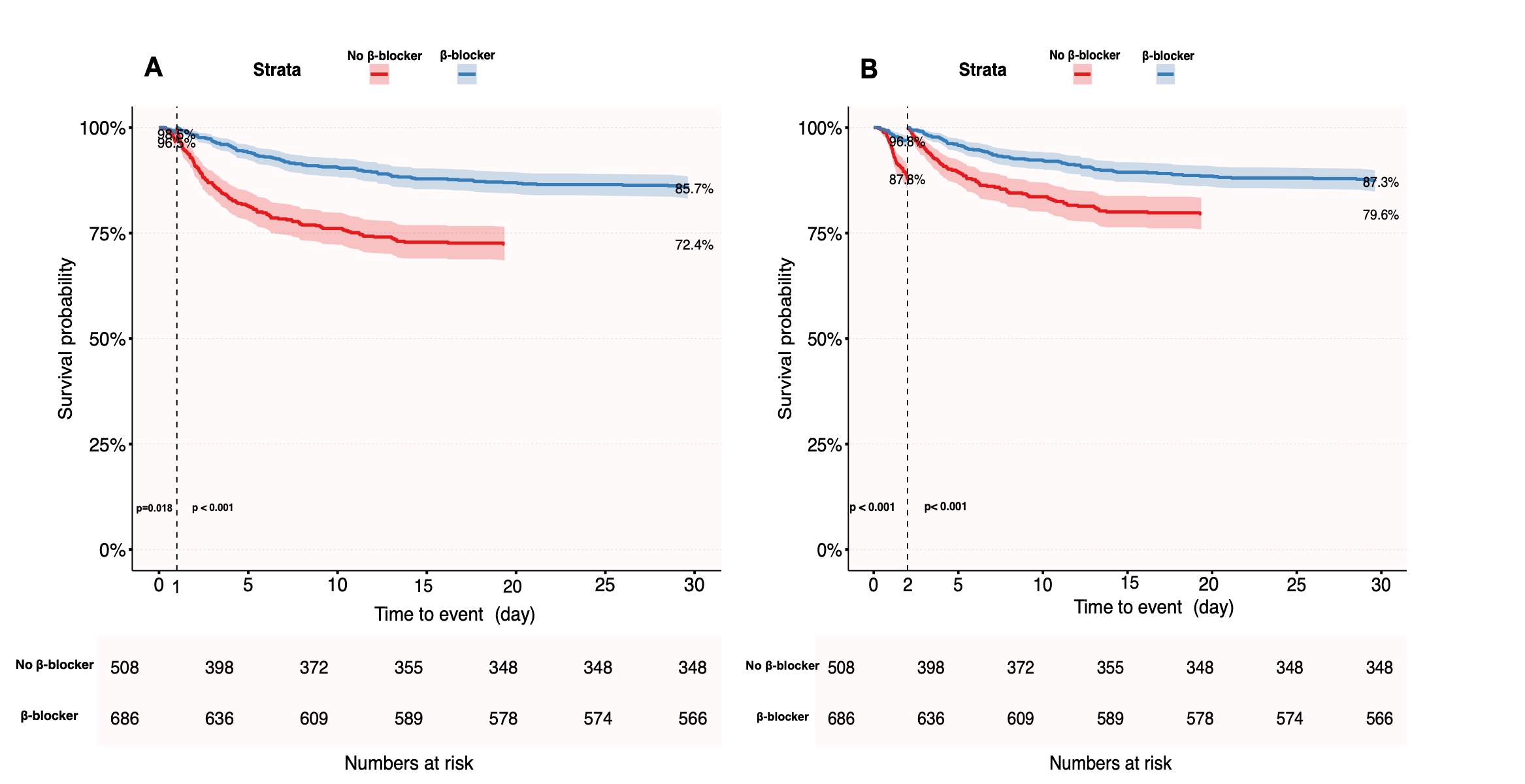

Supplement: Supplementary file 18 [file Image_1.jpeg]

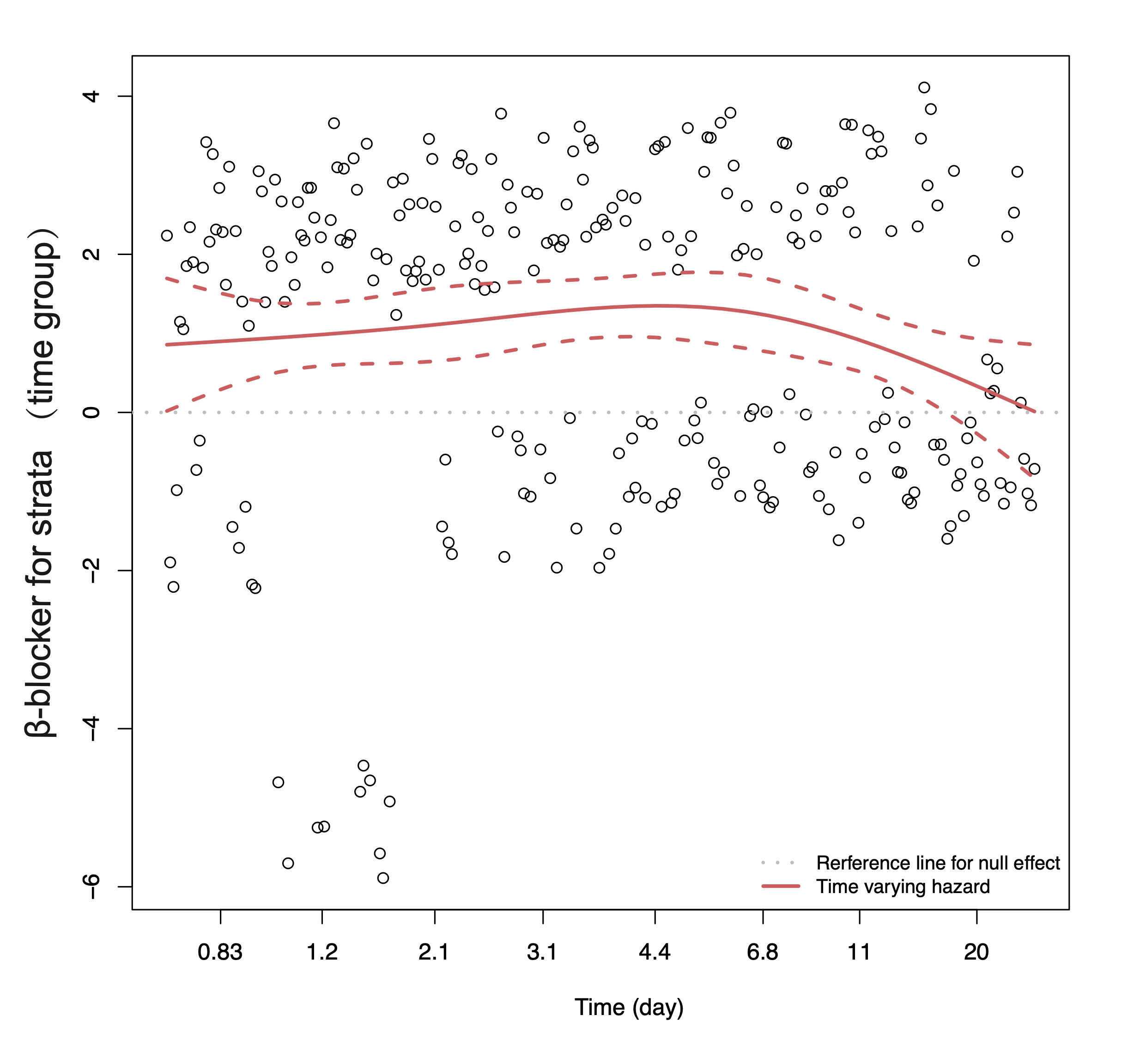

Supplement: Supplementary file 19 [file Image_2.jpeg]
