# Supplementary material for: Association between the use of β-adrenergic receptor blockers and all-cause mortality in sepsis-associated rhabdomyolysis syndrome: a cohort study
Source: Front Med (Lausanne). 2026 Feb 13;13:1743813. doi: 10.3389/fmed.2026.1743813 (PMC12946102; doi:10.3389/fmed.2026.1743813)
Supplement: Supplementary file 16 [file Data_Sheet_4.pdf]

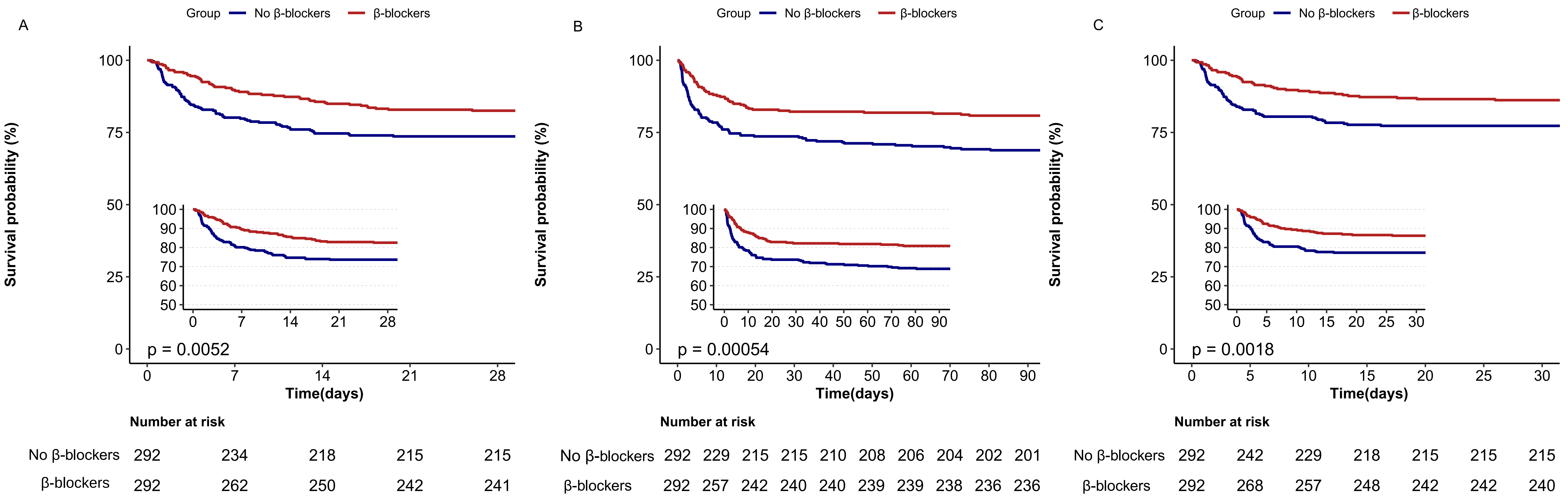
A
Group
No β-blockers
β-blockers
Survival probability (%)
100
75
50
25
0
p = 0.0052
0
7
14
21
28
Time(days)
Number at risk
No β-blockers 292 234 218 215 215
β-blockers 292 262 250 242 241
B
Group
No β-blockers
β-blockers
Survival probability (%)
p = 0.00054
0 10 20 30 40 50 60 70 80 90
Time(days)
Number at risk
No β-blockers 292 229 215 215 210 208 206 204 202 201
β-blockers 292 257 242 240 240 239 239 238 236 236
C
Group
No β-blockers
β-blockers
Survival probability (%)
p = 0.0018
0 5 10 15 20 25 30
Time(days)
Number at risk
No β-blockers 292 242 229 218 215 215 215
β-blockers 292 268 257 248 242 242 240
