# Supplementary material for: Association between the use of β-adrenergic receptor blockers and all-cause mortality in sepsis-associated rhabdomyolysis syndrome: a cohort study
Source: Front Med (Lausanne). 2026 Feb 13;13:1743813. doi: 10.3389/fmed.2026.1743813 (PMC12946102; doi:10.3389/fmed.2026.1743813)
Supplement: Supplementary file 17 [file Data_Sheet_5.pdf]

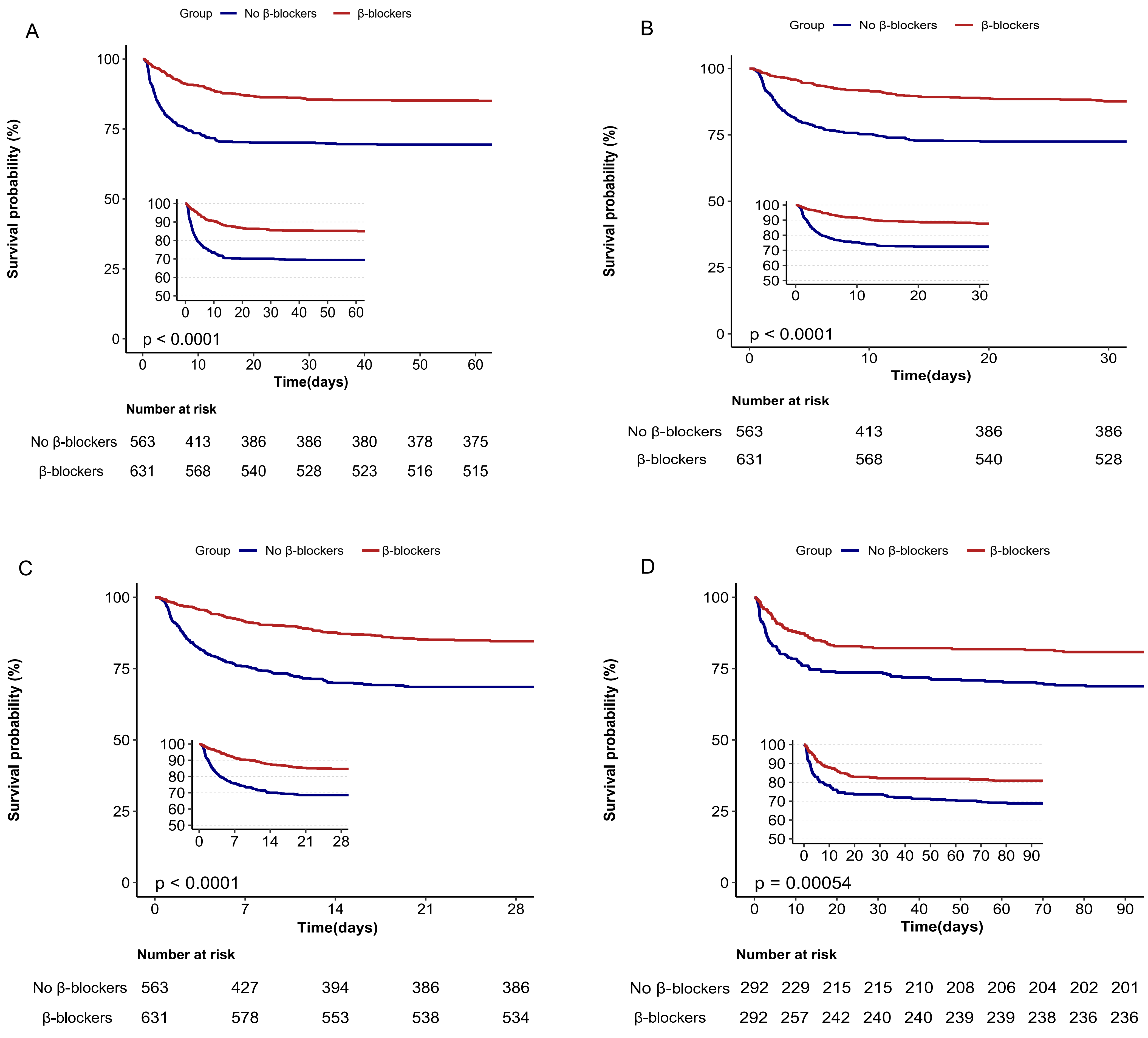
A
Group No β-blockers β-blockers
Survival probability (%)
100
75
50
25
0
p < 0.0001
0 10 20 30 40 50 60
Time(days)
Number at risk
No β-blockers 563 413 386 386 380 378 375
β-blockers 631 568 540 528 523 516 515
B
Group No β-blockers β-blockers
Survival probability (%)
p < 0.0001
0 10 20 30
Time(days)
Number at risk
No β-blockers 563 413 386 386
β-blockers 631 568 540 528
C
Group No β-blockers β-blockers
Survival probability (%)
p < 0.0001
0 7 14 21 28
Time(days)
Number at risk
No β-blockers 563 427 394 386 386
β-blockers 631 578 553 538 534
D
Group No β-blockers β-blockers
Survival probability (%)
p = 0.00054
0 10 20 30 40 50 60 70 80 90
Time(days)
Number at risk
No β-blockers 292 229 215 215 210 208 206 204 202 201
β-blockers 292 257 242 240 240 239 239 238 236 236
